# N-terminal oligomerization drives HDAC4 nuclear condensation and neurodevelopmental dysfunction in *Drosophila*

**DOI:** 10.1098/rsob.250095

**Published:** 2025-10-29

**Authors:** Hannah R. Hawley, Andrew J. Sutherland-Smith, Matthew S. Savoian, Helen L. Fitzsimons

**Affiliations:** ^1^ School of Food Technology and Natural Sciences, Massey University, Palmerston North, New Zealand

**Keywords:** *Drosophila*, brain, HDAC4, condensate, neuron, development, mushroom body, histone deacetylase, Alzheimer’s, MEF2

## Abstract

Histone deacetylase four (HDAC4) undergoes dynamic nucleocytoplasmic shuttling, a process critical for regulating its activity. However, aberrant nuclear accumulation of HDAC4 is associated with both neurodevelopmental and neurodegenerative disease, and in our *Drosophila* model, impairs normal neuronal development. Upon nuclear accumulation, HDAC4 forms biomolecular condensates, previously termed aggregates, that correlate with the severity of defects in development of the *Drosophila* mushroom body and adult eye. Here we determined that nuclear condensation of HDAC4 is dependent on self-oligomerization, and that impairing oligomerization reduces condensation and the severity of neurodevelopmental phenotypes in *Drosophila*. HDAC4 condensates are highly dynamic and are stabilized by the presence of MEF2, which promotes their formation, ultimately exacerbating phenotypic severity. These data provide insight into the role of HDAC4 condensates in normal neuronal function and suggest that their dysregulation may contribute to neurodevelopmental disorders. Consequently, targeting oligomerization of HDAC4 and its interaction with MEF2 present potential therapeutic strategies for diseases associated with HDAC4 nuclear accumulation.

**Figure:**
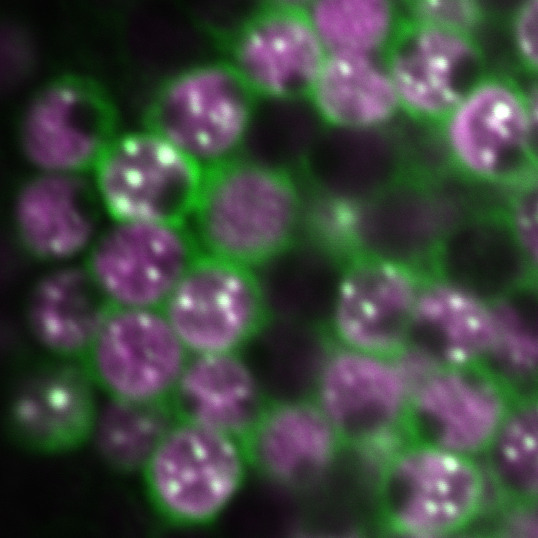

## 1. Introduction

Histone deacetylase four (HDAC4) is an important regulator of neuronal development and memory formation in species across the animal kingdom including *C. elegans*, *Drosophila*, rodents and humans [1–7]. In mammals, HDAC4 is expressed widely in the brain [8–10]. As a member of the Class IIa family of histone deacetylases, it is characterized by a conserved deacetylase domain and an extended N-terminal region that interacts with regulatory proteins including transcription factors [11–14]. Although it contains a deacetylase domain, vertebrate HDAC4 harbours little intrinsic deacetylase activity [15], instead facilitating deacetylation via association with HDAC3 and the NCoR/SMRT repressor complex [16–18].

HDAC4 shuttles between the nucleus and cytoplasm in a phosphorylation-dependent manner. Nuclear export is mediated by 14-3-3 binding to phosphorylated serines (Ser246, Ser467 and Ser632 in human HDAC4) [19–22], while nuclear import is mediated through interaction with the transcription factor MEF2 [14,23]. This dynamic regulation is governed by synaptic activity and differs between neuronal subtypes [3,5,9,24–27].

Aberrant nuclear accumulation of HDAC4 has been associated with several neurodegenerative and neurodevelopmental disorders. Increased nuclear concentration of HDAC4 has been observed in the brains of individuals with Alzheimer’s disease (AD) [28,29] and ataxia telangiectasia [30], as well as in mouse models of AD [28,31,32], CDKL5 disorder [7], 2q37 deletion syndrome [3] and Parkinson’s disease [33,34]. Mutations within the 14-3-3 binding site of HDAC4 have been identified in seven unrelated individuals with intellectual disability and developmental delay, and these mutations reduce the affinity of HDAC4 for 14-3-3 in cultured cells [6]. Consequently, it is hypothesized that disrupted nucleocytoplasmic shuttling of HDAC4 underlies neuronal dysfunction in these individuals.

HDAC4 staining in neurons displays a punctate, granular pattern [9,35], which is thought to involve self-oligomerization mediated by the N-terminal helix [36,37]. When HDAC4 accumulates in neuronal nuclei it forms larger punctate foci [32,34,38], which have been variously described as speckles or aggregates. Recent evidence indicates that HDAC4 foci are more accurately described as biomolecular condensates (termed condensates herein) [39], which are dynamic subnuclear compartments that regulate molecular interactions [40]. Disruption of these condensates, as observed in neuronal disease, is therefore likely to contribute to neuronal dysfunction. Despite numerous observations of nuclear HDAC4 accumulation in neuronal disease, the molecular mechanisms driving condensate formation and their contribution to disease onset and/or progression remain unexplored, highlighting a critical gap in understanding.

*Drosophila* HDAC4 shares key regulatory motifs with human HDAC4, including nuclear localization/export signals, the MEF2 binding region, catalytic site, ankyrin repeat binding region and conserved phosphorylation sites [4,35]. HDAC4 is highly expressed throughout the *Drosophila* brain [4,35], including in the mushroom body, which is a key integration centre for sensory information that shares many architectural features with the vertebrate cerebellum [41]. The intrinsic neurons of the mushroom body, known as Kenyon cells, exhibit nuclear condensate formation and impaired long-term memory upon expression of either human or *Drosophila* HDAC4 [4,42]. Developmental overexpression of HDAC4 disrupts both mushroom body and eye formation [35,42,43], and the severity is exacerbated by nuclear accumulation of HDAC4 [35,42].

The propensity of HDAC4 to form condensates appears to stem from its glutamine-rich N-terminus, as deletion of residues 1−179 of human HDAC4 prevents nuclear condensate formation [37]. This region mediates homo-oligomerization of HDAC4 through its N-terminal α-helix; in solution, the N-terminus of human HDAC4 forms an α-helix that self-associates in a four-helix bundle [36]. This tetramerization is dependent on the formation of a small hydrophobic core, consisting of Leu89, Ile90 and Phe93, which is supported by glutamine-dominated polar interaction networks. Substitution of Phe93 for a polar residue abolishes tetramerization in solution, while mutation of nearby His97 to phenylalanine extends the core and stabilizes tetramerization [36]. Furthermore, it was recently demonstrated that in the presence of DNA and MEF2, the N-terminal region of HDAC4 preferentially forms a dimer, with each HDAC4 molecule bound to a MEF2 dimer that associates with its cognate DNA molecule [44].

**Figure 1.**
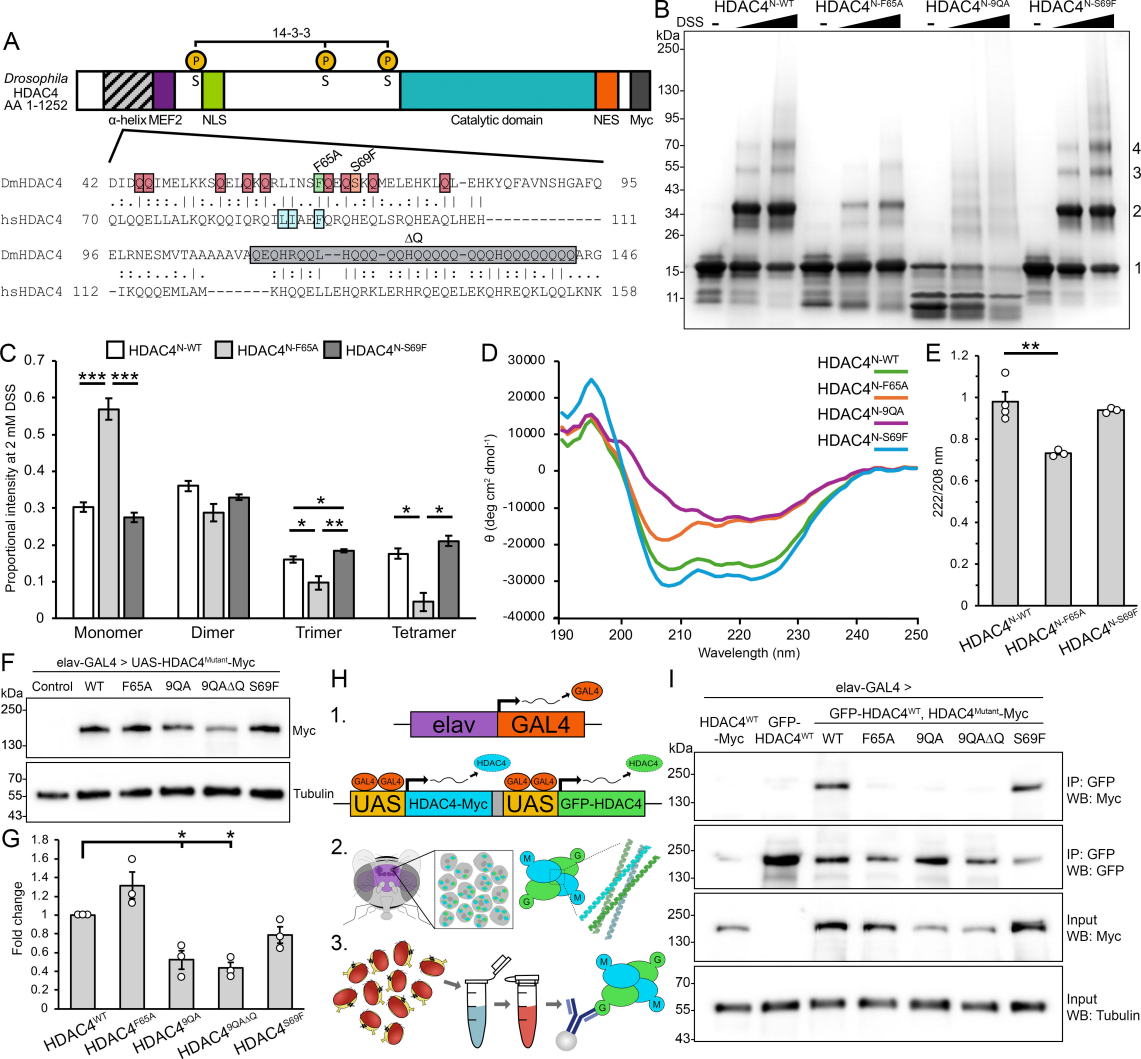
Mutation of the hydrophobic core or conserved glutamine residues alters oligomerization of DmHDAC4 in the adult brain. (A) Schematic diagram depicting the domain structure of *Drosophila* HDAC4 (top, DmHDAC4), including the N-terminal α-helix, MEF2 binding site (MEF2, purple) nuclear localization signal (NLS, green), 14-3-3 binding sites (P, phosphorylated serine (S) residues, yellow), catalytic domain (teal), nuclear export signal (NES, orange) and C-terminal Myc-tag (grey). Alignment of the α-helix region of hsHDAC4 and DmHDAC4 (bottom). Mutants used in this study: F65A, green box; 9QA, red boxes; 9QA∆Q, red boxes and grey ∆Q; S69F, orange box. In the 3SA mutant, the three phosphorylated serines (S) were substituted for alanines. (B) SDS-PAGE of chemically crosslinked purified recombinant wild-type and mutant N-terminal HDAC4 (HDAC4^N^). In the absence of crosslinker (–, DMSO only) HDAC4^N^ appears as an approximately 15 kDa monomer (1). Addition of 0.2 mM or 2.0 mM disuccinimidyl suberate (DSS) induces crosslinking, enabling visualization of dimer (2), trimer (3) and tetramer (4) species. (C) Quantification of HDAC4^N^ oligomerization following treatment with 2.0 mM DSS. The average proportion of monomer, dimer, trimer and tetramer species for each lane is displayed (*n* = 3 experiments). HDAC4^N-F65A^ is significantly reduced in trimer (*p* = 0.03091) and tetramer (*p* = 0.009762), and increased in monomer species compared with HDAC4^N-WT^ (*p* = 0.0002848). HDAC4^N-S69F^ is significantly increased in trimer species compared with HDAC4^N-WT^ (*p* = 0.0282). ANOVA: monomer *F*_(2,6)_ = 59.77, *p* < 0.0001; dimer *F*_(2,6)_ = 2.67, *p* = 0.1483; trimer *F*_(2,6)_ = 24.57, *p* = 0.001288; tetramer *F*_(2,6)_ = 15.51, *p* = 0.004256. *Post hoc* Tukey’s HSD, *p < 0.05, **p < 0.01, ***p < 0.001. Error bars indicate SEM. (D) Circular dichroism spectra of purified HDAC4^N^ variants. HDAC4^N-WT^ and HDAC4^N-S69F^ exhibit α-helical folding, with characteristic negative peaks at 208 and 222 nm. HDAC4^N-F65A^ shows a partial loss of α-helicity, while HDAC4^N-9QA^ exhibits a more pronounced reduction in α-helicity, indicating a shift towards a less ordered conformation. (E) 222/208 nm ratios from CD spectra of purified HDAC4^N^. HDAC4^N-WT^ and HDAC4^N-S69F^ maintain a ratio close to 1, consistent with oligomerization, whereas HDAC4^N-F65A^ shows a significant reduction, suggesting impaired oligomerization (*n* = 3 independent spectra). ANOVA, *F*_(2,7)_ = 13.77, *p* = 0.003747; *post hoc* Tukey’s HSD, **p < 0.01. Error bars indicate SEM. (F) Whole cell lysates generated from adult heads expressing HDAC4^WT^-Myc or HDAC4^Mutant^-Myc under the control of *elav-GAL4* were subjected to SDS-PAGE and probed for Myc and tubulin. The control is *elav-GAL4/+*. (G) Quantification of HDAC4 band intensity normalized to tubulin (*n* = 3 blots). HDAC4^9QA^ and HDAC4^9QA∆Q^ are significantly reduced compared with HDAC4^WT^. One sample *t*‐test compared with HDAC4^WT^: HDAC4^9QA^ *t*_(2)_ = 4.8747, *p* = 0.0396; HDAC4^9QA∆Q^ *t*_(2)_ = 9.1283, *p* = 0.0118. (H) Schematic of the co-immunoprecipitation workflow used in (I). 1. GFP- (green) and Myc-tagged (blue) HDAC4 are expressed in flies with *elav-GAL4*. 2. HDAC4 condensates comprise homo- and hetero-tetramers of GFP- and Myc-tagged HDAC4 in Kenyon cell nuclei. 3. GFP-HDAC4 is immunoprecipitated from whole cell lysates of adult fly heads and co-immunoprecipitated HDAC4-Myc (WT or mutant) is detected. (I) Genotypes were generated by crossing *elav-GAL4* females to males carrying the indicated *HDAC4* transgene. Flies were raised at 18°C until eclosion when adults were transferred to 22°C to increase transgene expression. Co-immunoprecipitation yield of HDAC4^F65A^ (lane 4), HDAC4^9QA^ (lane 5), and HDAC4^9QA∆Q^ (lane 6) is significantly reduced compared with HDAC4^WT^ (lane 3) upon immunoprecipitation of GFP-HDAC4.

While HDAC4 condensates have been recently documented [39], the mechanisms driving their formation and their relevance to neuronal dysfunction remain unclear. Here we aimed to determine whether HDAC4 oligomerization is required for condensate formation, and whether disrupting this process alters HDAC4-induced neurodevelopmental phenotypes in *Drosophila*. Using full-length HDAC4 mutants based on structural data, we demonstrate that oligomerization promotes nuclear import and condensate formation and determined that both oligomerization and MEF2 binding contributed to neurodevelopmental defects. Importantly, the level of HDAC4 overexpression resulting in these defects is modest at approximately 1.3-fold higher than endogenous levels [35], suggesting the phenotypes are not simply artefacts of high overexpression. This approach allows us to probe the molecular features that drive condensate formation and assess their functional relevance *in vivo* in a model with controlled and physiologically relevant expression levels. These findings offer insight into HDAC4 function, and how its dysregulation may contribute to neurological disease.

## 2. Results

### 2.1. Characterization of HDAC4 oligomerization mutants

We first sought to examine whether the mechanism of oligomerization of *Drosophila* HDAC4 (DmHDAC4) is conserved with human HDAC4 (hsHDAC4). The sequence of the N-terminal α-helix of hsHDAC4 is highly conserved with DmHDAC4 (figure 1A). Notably, the three non-polar residues that form the hydrophobic core (Leu61, Ile62 and Phe65 in *Drosophila*) are all strictly conserved, as are nine of the glutamine residues that stabilize intra- and interhelical interactions [36]. As *in vitro* tetramerization of hsHDAC4 is destabilized upon substitution of Phe93 within the hydrophobic core [36], we generated the homologous mutation within DmHDAC4, Phe65Ala (HDAC4^F65A^). Either side of this core are the glutamine residues that form intra- and interhelical polar interaction networks. To investigate the importance of these networks for tetramer formation, the nine conserved glutamine residues were substituted with alanines (HDAC4^9QA^). While the N-termini of both hsHDAC4 and DmHDAC4 are glutamine-rich, DmHDAC4 has a significant polyglutamine stretch C-terminal to the nine conserved glutamines. Given the role that polyglutamine regions have in protein aggregation [45], as well as the roles of glutamines in the potentially stabilizing interaction networks of the HDAC4 tetramer [36], this 32 amino acid stretch (residues Gln112–Gln143) containing 25 glutamine residues was deleted alongside the 9QA substitutions (HDAC4^9QA∆Q^) to determine whether it confers additional stability to DmHDAC4 oligomers. Conversely, since hsHDAC4 tetramers are stabilized by extension of the hydrophobic core via substitution of His97 to Phe [36], we also introduced a hydrophobic Phe at the aligned position in *Drosophila* HDAC4 (HDAC4^S69F^).

#### 2.1.1. *In vitro* oligomerization

To characterize the capacity of these mutants to oligomerize, *in vitro* chemical crosslinking experiments were performed using purified recombinant N-terminal fragments spanning Pro37 to Gln143 of DmHDAC4, which corresponds to the region of hsHDAC4 examined by [36]. To avoid confusion with full-length HDAC4 constructs, these are denoted as HDAC4^N-mutant^. HDAC4^N-WT^ formed oligomers corresponding to homodimer, trimer and tetramers as well as higher order species (figure 1B). HDAC4^N-F65A^ showed a significant reduction in trimer and tetramer species and was increased in monomer species compared with HDAC4^N-WT^, whereas HDAC4^N-S69F^ exhibited a significant increase in trimers (figure 1C). HDAC4^N-9QA^ behaved markedly differently—expression in *E. coli* was notably inefficient, requiring approximately 100-fold more culture volume to achieve comparable yields to HDAC4^N-WT^, and significant degradation occurred during affinity and size-exclusion purification. Crosslinking and SDS-PAGE analysis revealed a smear rather than discrete oligomeric species, indicating these degradation products are capable of oligomerization. Changes in oligomerization between the mutants and wild-type were further supported by shifts in retention volume during size-exclusion chromatography (electronic supplementary material, figure S1), consistent with findings for human HDAC4 [44]. Collectively, these results show that disruption of the hydrophobic core in HDAC4^N-F65A^ decreases oligomer formation with no impact on protein stability, whereas mutation of the glutamine residues in HDAC4^N-9QA^ severely compromises protein stability and proper oligomer formation.

Circular dichroism analysis revealed that HDAC4^N-WT^ primarily adopted an α-helical secondary conformation in solution, similarly to hsHDAC4 [36], as did HDAC4^N-F65A^ and HDAC4^N-S69F^ (figure 1D). However, features of α-helix formation were lost for HDAC4^N-9QA^, likely due to its inherent instability and the high concentration of degradation products. Comparison of the 222/208 nm ratio for structures that form an α-helix provides insight into helical packing and oligomerization of proteins, where ratios close to or above 1 suggest coiled-coil or tightly packed helices, and lower values indicate more isolated helices or structural destabilization [46]. Both HDAC4^N-WT^ and HDAC4^N-S69F^ had ratios close to 1 (figure 1E), supporting that they exist as oligomeric helices, while the ratio for HDAC4^N-F65A^ was reduced, suggestive of forming more isolated helices. These data indicate that the substitution of Phe65 to Ala destabilizes oligomerization, whereas it is stabilized by the substitution of Ser69 to Phe. Moreover the glutamine residues are required for the correct α-helical folding of HDAC4.

#### 2.1.2. *In vivo* oligomerization

To characterize the ability of each of the full-length HDAC4 mutants to oligomerize *in vivo*, transgenic flies were generated carrying UAS-HDAC4^WT^ or each of the mutants (hereafter collectively referred to as HDAC4^Mutant^) for UAS/GAL4 regulated expression in the brain [47]. We adopted an overexpression system using the pan-neuronal *elav-GAL4* driver [48,49] for phenotypic analysis. In a previous study, we compared endogenous and transgene HDAC4 via western blot [35]. Based on quantification of those data, we determined that HDAC4 levels are elevated by approximately 1.3-fold over endogenous levels, indicating modest overexpression. The expression levels of HDAC4^F65A^ and HDAC4^S96F^ were not significantly different from those of HDAC4^WT^. By contrast, HDAC4^9QA^ and HDAC4^9QA∆Q^ were expressed at approximately half the level of HDAC4^WT^ (figure 1F,G; *p* < 0.001). This reduction in protein level is consistent with the biochemical evidence showing instability of HDAC4^9QA^ and suggests that, in addition to mediating oligomerization and stabilizing the N-terminal helix of HDAC4, the N-terminal glutamine residues contribute to the proper folding and stability of full length HDAC4 *in vivo*.

To determine whether the HDAC4 mutants retained the capacity to oligomerize, co-immunoprecipitation (co-IP) was performed on whole head lysates from flies in which UAS-GFP-HDAC4^WT^ [42] was pan-neuronally co-expressed with either UAS-HDAC4^WT^-Myc or UAS-HDAC4^Mutant^-Myc via the *elav-GAL4* driver (figure 1H). Immunoprecipitation of GFP-HDAC4^WT^ led to robust co-IP of HDAC4^WT^-Myc (figure 1I, lane 3), confirming oligomerization of wild-type DmHDAC4. HDAC4^F65A^, HDAC4^9QA^ and HDAC4^9QA∆Q^ showed a marked reduction in co-IP (figure 1G, lanes 4−6), revealing a severely compromised ability to oligomerize. However, given that HDAC4^9QA^ and HDAC4^9QA∆Q^ were expressed at approximately half that of HDAC4^WT^, it is unclear whether the reduction in co-IP efficiency is a result of lower abundance and/or a change in oligomerization.

For the S69F mutation, which enhanced crosslinking *in vitro* (figure 1C), co-IP with GFP-HDAC4^WT^ was similar to HDAC4^WT^. However, the amount of GFP-HDAC4^WT^ recovered by immunoprecipitation was consistently reduced in the presence of HDAC4^S69F^, leading to a modest increase in the ratio of co-IPed HDAC4^S69F^-Myc relative to the GFP-HDAC4^WT^ input (figure 1I, lane 7). This could suggest more efficient incorporation of HDAC4^S69F^ into oligomers; however, this should be interpreted cautiously as co-IP efficiency may also be influenced by complex stoichiometry involving additional binding partners or saturation effects.

Together, these findings indicate that both the hydrophobic core and glutamine-mediated polar interaction networks and/or α-helical folding are essential for N-terminal oligomerization of HDAC4 *in vitro*, and for the self-association of full-length HDAC4 *in vivo*. For clarity, we refer to this multimerization as oligomerization, although the precise stoichiometry of HDAC4 assemblies *in vivo* (e.g. dimers, trimers or higher-order species) has not yet been resolved.

### 2.2. Oligomerization of HDAC4 correlates with nuclear condensate formation in mushroom body neuronal nuclei

**Figure 2.**
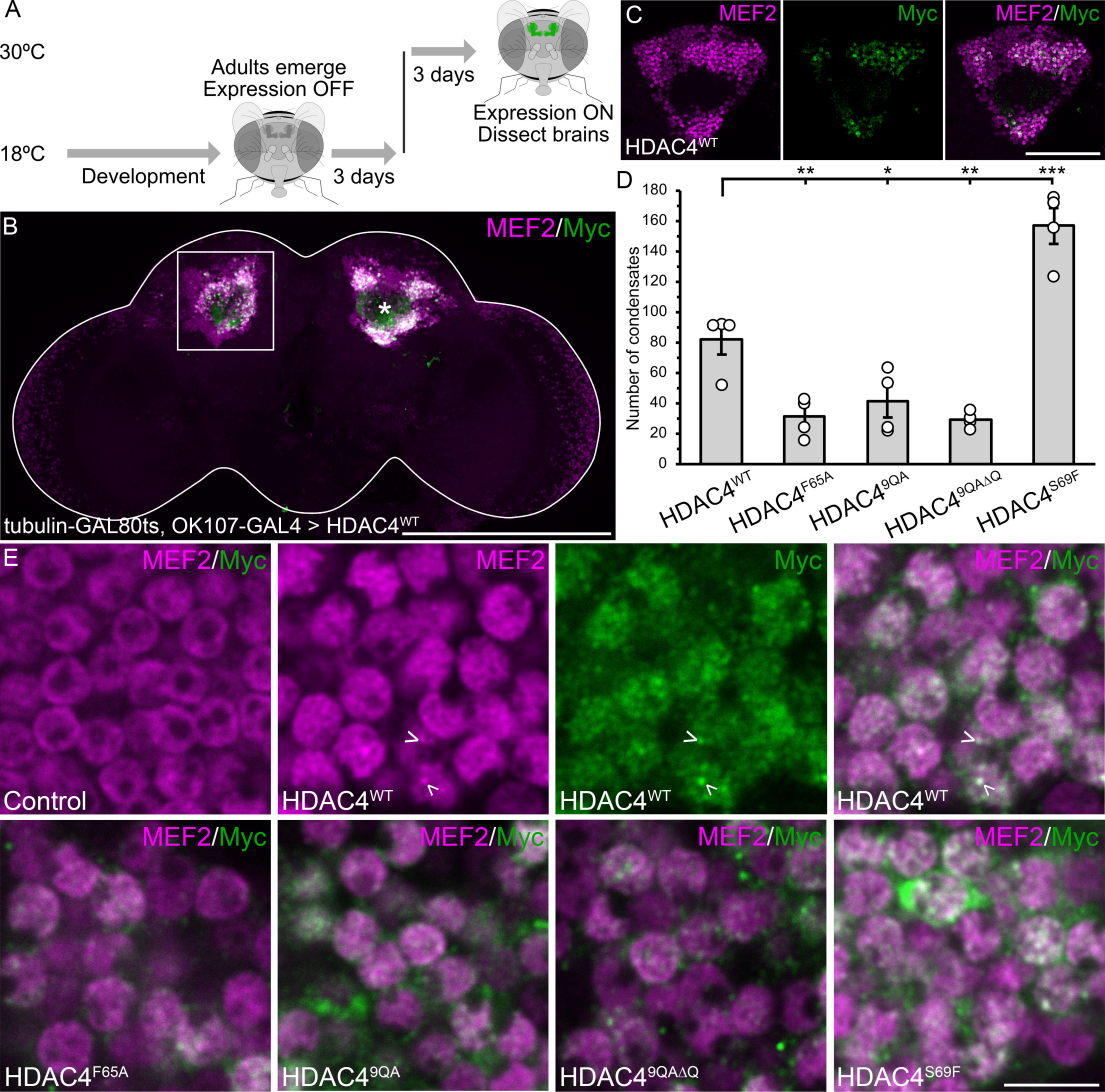
HDAC4 oligomerization capacity correlates with formation of nuclear condensates. All genotypes were generated by crossing *tubP-GAL80ts;OK107-GAL4* females to males carrying the indicated *UAS-HDAC4-Myc* transgene or the *w*(*CS10*) control. Transgene expression was induced in adults by shifting flies from 18°C (GAL80ts active) to 30°C (GAL80ts inactive) after eclosion. Brains were dissected 72 h post-induction. (A) Schematic of adult-specific gene induction protocol. (B,C,E) Whole mount brains stained with anti-Myc (green) and anti-MEF2 (magenta). (B) Maximum projection showing HDAC4^WT^-Myc expression, which colocalizes with endogenous MEF2 in Kenyon cell nuclei. Asterisk labels the calyx and the boxed region is depicted in (C). Scale bar = 200 µm. (C) Single optical section (0.5 µm) through the calyx and Kenyon cell layer showing HDAC4^WT^ colocalization with MEF2. Scale bar = 50 µm. (D) Quantification of nuclear condensates (colocalizing HDAC4/MEF2 puncta) per 1 µm section (as shown in C) (*n* = 4 brains per genotype, 10 sections per brain). ANOVA, *F*_(4,15)_ = 36.88, *p* < 0.0001; *post hoc* Tukey’s HSD, *p < 0.05, **p < 0.01, ***p < 0.001. Error bars indicate SEM. (E) Representative 0.5 µm sections showing HDAC4 condensates (arrowheads). Scale bar = 5 µm.

We next examined whether the impaired ability of HDAC4 to oligomerize led to reduced nuclear condensate formation *in vivo*. At low expression levels, HDAC4 condensates are present but difficult to visualize reliably due to weaker signal intensity. Therefore, higher expression was necessary to achieve robust and consistent detection of condensates across cells, enabling accurate quantification. To that end, we drove expression of UAS-HDAC4-Myc with the stronger mushroom body driver *OK107-GAL4* [48,50]. To ensure that transgene expression did not impact the development of these cells, expression was induced in the adult brain via the TARGET system in which GAL4 is repressed during development at 18°C by a temperature-sensitive mutant of GAL80 (GAL80ts). Once adult flies emerged, they were transferred to 30°C, at which temperature GAL80ts repression of GAL4 is relieved and HDAC4 transgene expression is permitted [51] (figure 2A). MEF2 was used as a counterstain to enhance the visibility of HDAC4 condensates as it is sequestered in HDAC4 nuclear foci [4,12,35,52]. HDAC4^WT^ was robustly expressed in adult Kenyon cells (figure 2B,C), and formed nuclear condensates that colocalized with MEF2 (figure 2E), as observed previously [4,35]. Expression of HDAC4^F65A^, HDAC4^9QA^ and HDAC4^9QA∆Q^ resulted in significantly fewer condensates than HDAC4^WT^, whereas HDAC4^S69F^ expression doubled the number of condensates (figure 2D). These data collectively demonstrate a positive correlation between the N-terminal α-helix’s ability to oligomerize and the formation of condensates *in vivo*. Although condensate formation was significantly decreased by HDAC4^F65A^, HDAC4^9QA^ and HDAC4^9QA∆Q^, it was not completely abolished, likely due to the incomplete loss of oligomerization in each mutant (figure 1B). Additionally, oligomerization with endogenous HDAC4, which we have previously shown can form condensates with transgene HDAC4^WT^ [35], may also contribute to the observed condensate formation.

### 2.3. HDAC4 forms dynamic condensates *in vivo*

**Figure 3.**
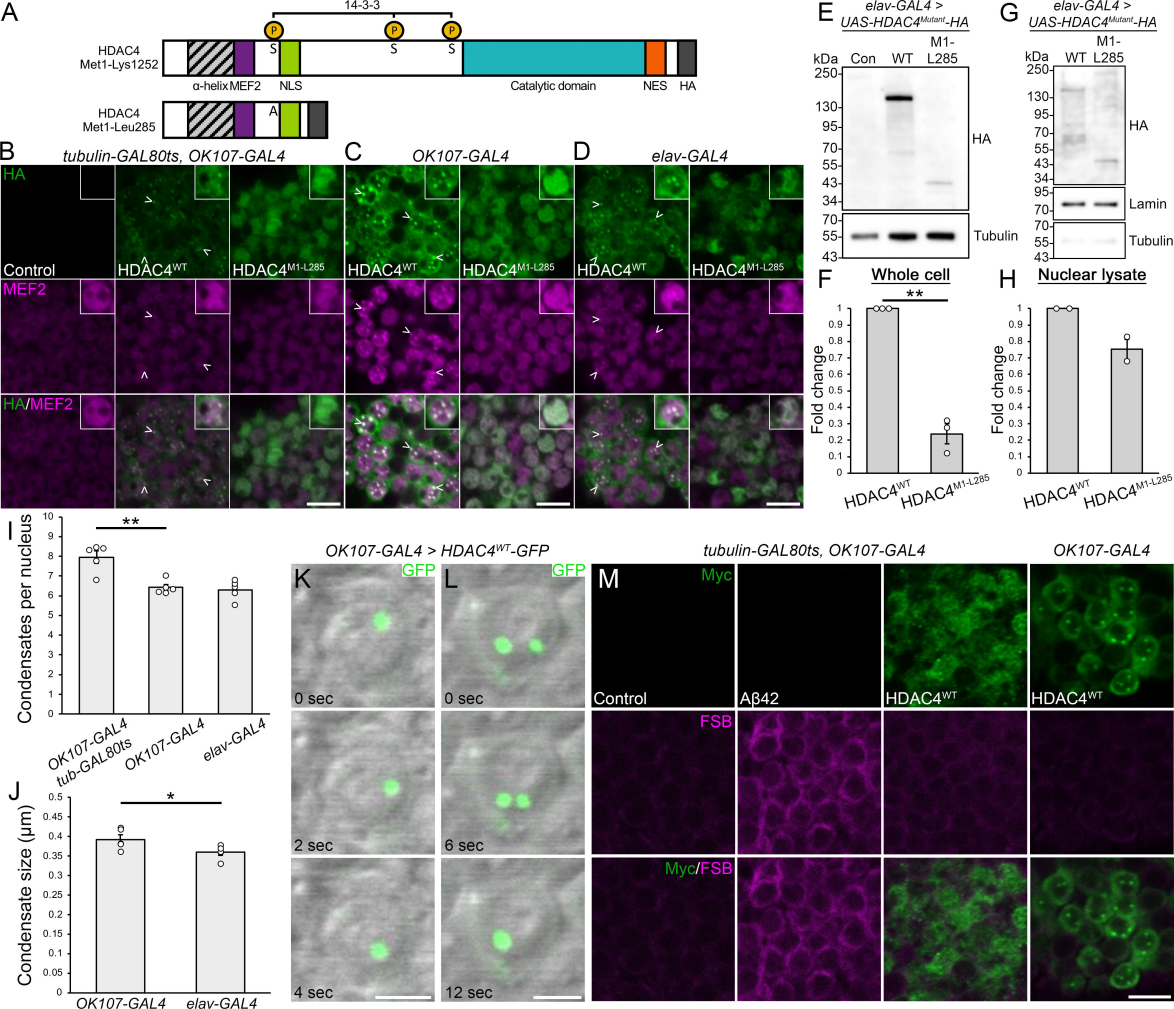
HDAC4 condensate formation requires regions downstream of the N-terminus and is dynamic. (A) Schematic diagram depicting the domain structure of full-length DmHDAC4 (top) and the truncated HDAC4^M1-L285^ (bottom). Ser239 is mutated to Ala in HDAC4^M1-L285^. (B–D) Representative 0.5 µm optical sections through the Kenyon cell layer stained with anti-Myc (green) and anti-MEF2 (magenta). Scale bar = 5 µm. Insets show individual nuclei. (B) Expression of *UAS-HDAC4^WT^-HA* or *UAS-HDAC4^M1-L285^-HA* driven by *OK107-GAL4* and induced in adulthood with GAL80ts (30°C, 72 h). The control is *tubulin-GAL80ts/+;OK107-GAL4/+*. (C) Expression of *UAS-HDAC4^WT^-HA* or *UAS-HDAC4^M1-L285^-HA* driven by *OK107-GAL4* at 25°C. (D) Expression of *UAS-HDAC4^WT^-HA* or *UAS-HDAC4^M1-L285^-HA* driven by *elav-GAL4* at 25°C. (E) Western blot of HDAC4^WT^ or HDAC4^M1-L285^ in whole cell lysates generated from adult heads expressing HDAC4^WT^ or HDAC4^M1-L285^ under the control of *elav-GAL4*. The control (con) is *elav-GAL4/+*. (F) Quantification of HDAC4 band intensity (from E) normalized to tubulin (*n* = 3). HDAC4^M1-L285^ is significantly reduced compared with HDAC4^WT^. One sample *t*‐test, *t*_(2)_ = 12.633, *p* = 0.0062. Error bars indicate SEM. (G) Subcellular fractionation and western blotting was performed on fly heads. Membranes were probed with anti-HA, as well as anti-lamin and anti-tubulin to assess fractionation efficacy. (H) Quantification of nuclear HDAC4 (from G), normalized to lamin (*n* = 2). There is no significant difference in nuclear abundance between HDAC4^WT^ and HDAC4^M1-L285^. One sample *t*‐test, *t*_(1)_ = 3.348, *p* = 0.185. Error bars indicate SEM. (I) Quantification of HDAC4^WT^ nuclear condensation. The number of condensates (colocalizing puncta of HDAC4 and MEF2) per nucleus were counted and averaged for *n* ≥ 28 nuclei per section for *n* = 5 brains per genotype. ANOVA, *F*_(2,12)_ = 14.67, *p* = 0.000599; *post hoc* Tukey’s HSD, **p < 0.01. Error bars indicate SEM. (J) Quantification of HDAC4^WT^ condensate size. Condensate size was measured and averaged for *n* ≥ 257 condensates per section for *n* = 5 brains per genotype. Unpaired *t*‐test, *t*_(8)_ = 2.315, *p* = 0.0493. Error bars indicate SEM. (K,L) Live imaging of whole mount brains. Genotypes were generated by crossing *OK107-GAL4* females to *UAS-HDAC4^WT^-GFP* males, and raised at 18°C throughout development and adulthood. Differential interference contrast (DIC) microscopy was used to visualize nuclei (grey) and intrinsic GFP fluorescence was detected (green). (K) HDAC4^WT^-GFP condensates are mobile within nuclei. (L) Coalescence of two HDAC4^WT^-GFP condensates. Scale bar = 2 µm. (M) Anti-Myc (green) immunohistochemistry on whole brains stained with 1-fluoro-2,5-bis(3-carboxy-4-hydroxystyryl)benzene (FSB, magenta). Representative single optical sections (0.5 µm) through the Kenyon cell layer are shown. Genotypes were generated as described in (B,C). Scale bar = 5 µm.

Our data demonstrate that oligomerization via the N-terminus is required for nuclear condensation of HDAC4. However, it remained unclear whether other regions of the protein contribute to this process. To investigate this, we compared full-length HA-tagged HDAC4 (HDAC4^WT^) with a truncated N-terminal construct of HDAC4 comprising residues Met1 to Leu285 (HDAC4^M1-L285^). This region includes the MEF2 binding site to facilitate nuclear import, and one of the key 14-3-3 binding sites (Ser 239), which we mutated to alanine to prevent nuclear exit (figure 3A). When expressed in adult Kenyon cells under the same conditions as in figure 2, HDAC4^WT^ was observed in both cytoplasmic haloes and in the nucleus, where it formed condensates (figure 3B), whereas HDAC4^M1-L285^ localized exclusively to the nucleus, where it appeared more uniform and granular than HDAC4^WT^. This lack of condensation persisted even under constitutive expression with *OK107-GAL4* and *elav-GAL4* throughout development, which facilitates prolonged accumulation (figure 3C,D). These observations indicate that while the N-terminal region enables oligomerization, additional sequences within the C-terminus are necessary for condensate formation. To confirm that this was not a result of differing protein levels in the nucleus, we assessed HDAC4 levels by immunoblotting. While total HDAC4^WT^ levels were four times higher than HDAC4^M1-L285^ in whole head lysates (figure 3E,F), both had comparable levels in nuclear fractions (figure 3G,H), indicating that the inability of HDAC4^M1-L285^ to form condensates is not due to reduced nuclear concentration. These data also validate that condensate formation is not due to the presence of the Myc tag, and further we have also observed condensates with FLAG-tagged [4] and GFP-tagged [42] HDAC4 constructs.

We also observed that constitutive expression of HDAC4^WT^ using *OK107-GAL4* resulted in significantly fewer condensates per nucleus compared with Kenyon cells where expression was restricted to the adult brain using *tubulin-GAL80ts;OK107-GAL4* (figure 3I). However, the condensates in the constitutive expression conditions were larger than those resulting from *tubulin-GAL80ts;OK107-GAL4* expression, which were smaller than the diffraction limit and therefore not quantified. Furthermore, expression with *OK107-GAL4* resulted in larger condensates than *elav-GAL4* (figure 3J)*,* consistent with stronger expression driven by *OK107-GAL4* in Kenyon cells [48]. These observations suggest that, over time, condensates undergo fusion or coalescence, resulting in fewer but larger structures. To explore the dynamics of these structures, we performed live-imaging of HDAC4^WT^-GFP in mushroom body neurons. HDAC4 condensates displayed dynamic movement (137 out of 174 were mobile during a 100 s period) (figure 3K). Although fusion events were infrequent (three observed during the 100 s period) they did occur (figure 3L); therefore, HDAC4 forms dynamic condensates *in vivo* that can coalesce under certain circumstances.

Finally, we examined colocalization of HDAC4 condensates with the Congo red derivative FSB (1-fluoro-2,5-bis(3-carboxy-4-hydroxystyryl)benzene) and thioflavin-T (THT), markers of β-sheet amyloid aggregation [53]. FSB and THT successfully detected the 42 amino acid Aβ fragment of human Amyloid Precursor Protein in Kenyon cells when expressed under the control of *OK107-GAL4* (figure 3M; electronic supplementary material, figure S2). However, neither FSB nor THT colocalized with HDAC4^WT^ condensates, suggesting that they are not dominated by amyloid cross β-sheet structures.

These findings highlight that HDAC4 condensate formation depends on both N-terminal oligomerization and C-terminal protein domains. This condensation occurs in a dynamic manner, influenced by expression level and duration, and lacks amyloid-like characteristics, suggesting that HDAC4 oligomerization and accumulation may have significant biological relevance. We continue to refer to HDAC4 inclusions as biomolecular condensates because their formation requires N-terminal self-association, and they exhibit hallmarks consistent with liquid–liquid phase separation (LLPS). These hallmarks include concentration-dependent foci formation and the ability to form into larger structures over time, features commonly observed in phase-separated condensates [54]. Furthermore, the involvement of intrinsically disordered domains and multivalent interactions within HDAC4 is consistent with LLPS-mediated assembly.

### 2.4. Condensation of HDAC4 correlates with the severity of HDAC4 overexpression-induced neurodevelopmental defects

**Figure 4.**
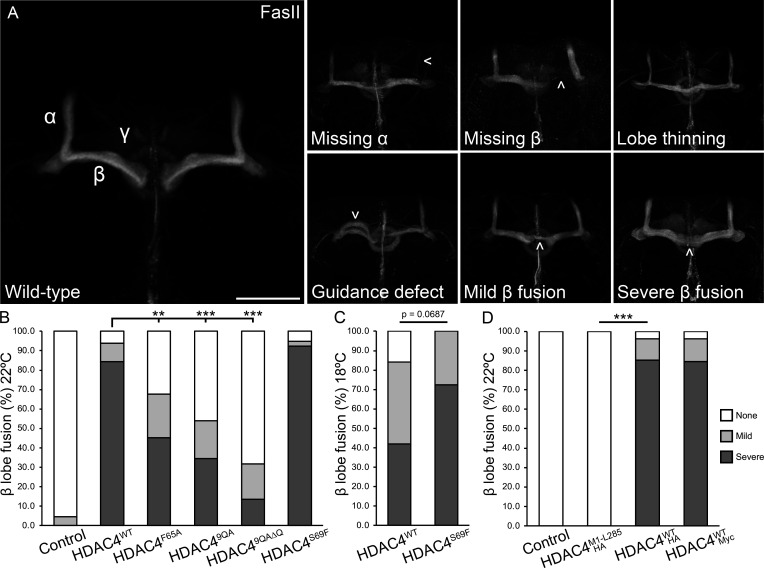
HDAC4 oligomerization correlates with severity of defects in mushroom body development. (A) Immunohistochemistry with anti-fasciclin II (FasII) labels the alpha (α), beta (β) and gamma (γ) lobes of the adult mushroom body. Maximum projections were generated from stacks acquired at 1 µm increments. Defects (arrows) resulting from HDAC4-overexpression include missing lobes, α and β lobe thinning, guidance defects, mild β lobe fusion, and severe β lobe fusion. Scale bar = 50 µm. (B–D) Quantification of β lobe fusion resulting from HDAC4 overexpression. All genotypes were generated by crossing *elav-GAL4* females to males carrying the indicated *UAS-HDAC4* transgene or the *w*(*CS10*) control. Mild fusion was counted when less than half of the β lobe was fused, and severe when fusion was greater than half. Significance was determined using Fisher’s exact test, **p < 0.01, ***p < 0.001. (B) The proportion of brains displaying β lobe fusion was significantly reduced for HDAC4^F65A^, HDAC4^9QA^ and HDAC4^9QA∆Q^ mutants compared with HDAC4^WT^. (C) At lower expression levels, the difference in severity between HDAC4^WT^ and HDAC4^S69F^ appeared larger but this was not statistically significant. (D) HDAC4^WT^-Myc and HDAC4^WT^-HA both induced β lobe fusion to a similar degree, while no fusion was observed for HDAC4^M1-L285^-HA.

Building on these findings, we next examined whether the capacity of each mutant to form condensates was linked to the severity of neurodevelopmental defects induced by HDAC4 in the mushroom body. The *Drosophila* mushroom body is a bilateral structure composed of bundled axons and thus is ideal for monitoring gross changes in axon morphogenesis. These axons project from Kenyon cell bodies in the posterior of the brain in a bundle termed the pedunculus before bifurcating based on their subtype (α/β, α′/β′ and γ) to form characteristic lobes (figure 4A) [55]. The cell adhesion molecule fasciclin II (FasII) is abundantly expressed in the α/β and γ lobes, allowing for clear visualization of these lobes when immunostained with anti-FasII [56]. Consistent with previous findings [35,42], expression of HDAC4^WT^-Myc with the pan-neuronal *elav-GAL4* driver resulted in various mushroom body defects including premature lobe termination (missing lobe), lobe thinning and lobe fusion (figure 4A, table 1).

**Table 1.** HDAC4 oligomerization mutants reduce HDAC4-overexpression-induced defects in mushroom body development. All genotypes were generated by crossing *elav-GAL4* females to males carrying each *UAS-HDAC4-Myc* transgene and to the *w*(*CS10*) control. The percentage of brains displaying each phenotype was calculated from the total number of brains analysed for each genotype (*n*). Statistical analysis was performed with Fisher’s exact test. Overexpression of HDAC4^WT^ resulted in a significant number of brains with β lobe fusion compared with control (*p* < 0.0001). Expression of HDAC4^F65A^ significantly reduced β lobe fusion compared with HDAC4^WT^ (*p* = 0.0017), as did expression of HDAC4^9QA^ (*p* < 0.0001) and HDAC4^9QA∆Q^ (*p* < 0.0001). Percentages may not sum to 100 due to rounding.

	22°C						18°C	
(% of brains)	control	HDAC4^WT^	HDAC4^F65A^	HDAC4^9QA^	HDAC4^9QA∆Q^	HDAC4^S69F^	HDAC4^WT^	HDAC4^S69F^
α lobe thinning	0	6	0	8	0	26	5	10
α lobe missing	0	0	0	4	0	5	0	0
β lobe thinning	0	3	0	8	0	13	11	3
β lobe missing	0	0	0	0	0	3	5	0
β lobe fusion	5	94	68	54	32	95	84	100
mild	5	9	23	19	18	3	42	28
severe	0	84	45	35	14	92	42	72
guidance defect	0	3	0	0	4	0	5	0
no defects	95	3	32	42	64	3	11	0
*n*	44	32	31	26	22	39	19	29

The most prominent defect was β lobe fusion, which results from a failure of the β lobe axons to correctly terminate, resulting in erroneous crossing of the midline. β lobe fusion was observed in the majority of HDAC4^WT^ brains, but was significantly reduced in HDAC4^F65A^, HDAC4^9QA^ and HDAC4^9QA∆Q^ brains (figure 4B, table 1). Subclassification of fusion severity to either mild or severe revealed that these mutant forms also reduced the severity of the phenotype (table 1). It should be noted that as HDAC4^9QA^ and HDAC4^9QAΔQ^ protein levels were only half that of HDAC4^WT^ and HDAC4^F65A^, the decreased phenotype may be attributable to their lower expression levels, as we have previously observed that the severity of β lobe fusion correlates with the level of HDAC4 expression [35]. Since neither HDAC4^9QA^ nor HDAC4^F65A^ completely eliminated condensation or mushroom body defects, we combined HDAC4^9QA^ and HDAC4^F65A^ mutations as we hypothesized this may further destabilize oligomerization and subsequent HDAC4 condensation; however, HDAC4^9QA-F65A^ did not further reduce β lobe fusion (electronic supplementary material, table S1, figure S3).

Given the almost complete penetrance of β lobe fusion resulting from expression of HDAC4^WT^, it was unsurprising that expression of HDAC4^S69F^ did not result in a further increase. As β lobe fusion severity correlates with the level of expression [35], we reduced GAL4 activity by lowering the temperature [57], to detect more subtle differences. Under these conditions, β lobe fusion remained near complete in HDAC4^S69F^ brains (100% total, 72% severe), and was reduced for HDAC4^WT^ (84% total, 42% severe), although not significantly (*p* = 0.0687) (table 1, figure 4C). Nevertheless, together these data together demonstrate a correlation between nuclear condensation of HDAC4 and the severity of β lobe fusion in the mushroom body.

We also confirmed that the observed phenotypes are not dependent on epitope tag choice, as expression of HDAC4^WT^-HA mirrored the defects seen with HDAC4^WT^-Myc (table 2). By contrast, expression of the N-terminal HDAC4^M1–L285^ fragment, which includes the oligomerization domain but lacks the C-terminal region, did not induce any detectable mushroom body defects (table 2, figure 4D), indicating that while the N-terminal region is required for condensation, it is not sufficient to disrupt mushroom body development. These data therefore support a model in which nuclear condensation of full-length HDAC4 is associated with, and likely contributes to, its deleterious effects in the developing brain.

**Table 2.** Full-length HDAC4 overexpression induces defects in mushroom body development. All genotypes were generated by crossing *elav-GAL4* females to males carrying each *UAS-HDAC4* transgene and to the *w*(*CS10*) control. Flies were raised at 22°C. The percentage of brains displaying each phenotype was calculated from the total number of brains analysed for each genotype (*n*). Statistical analysis was performed with Fisher’s exact test. Overexpression of HDAC4^WT^-Myc and HDAC4^WT^-HA each induced severe β lobe fusion (*p* < 0.0001).

	22°C			
(% of brains)	control	HDAC4^M1-L285^-HA	HDAC4^WT^-HA	HDAC4^WT^-Myc
α lobe thinning	4	0	59	35
α lobe missing	0	0	0	4
β lobe thinning	0	0	33	0
β lobe missing	0	0	0	4
β lobe fusion	0	0	97	96
mild	0	0	12	11
severe	0	0	85	85
guidance defect	0	0	4	0
no defects	100	100	0	4
*n*	23	22	27	26

**Figure 5.**
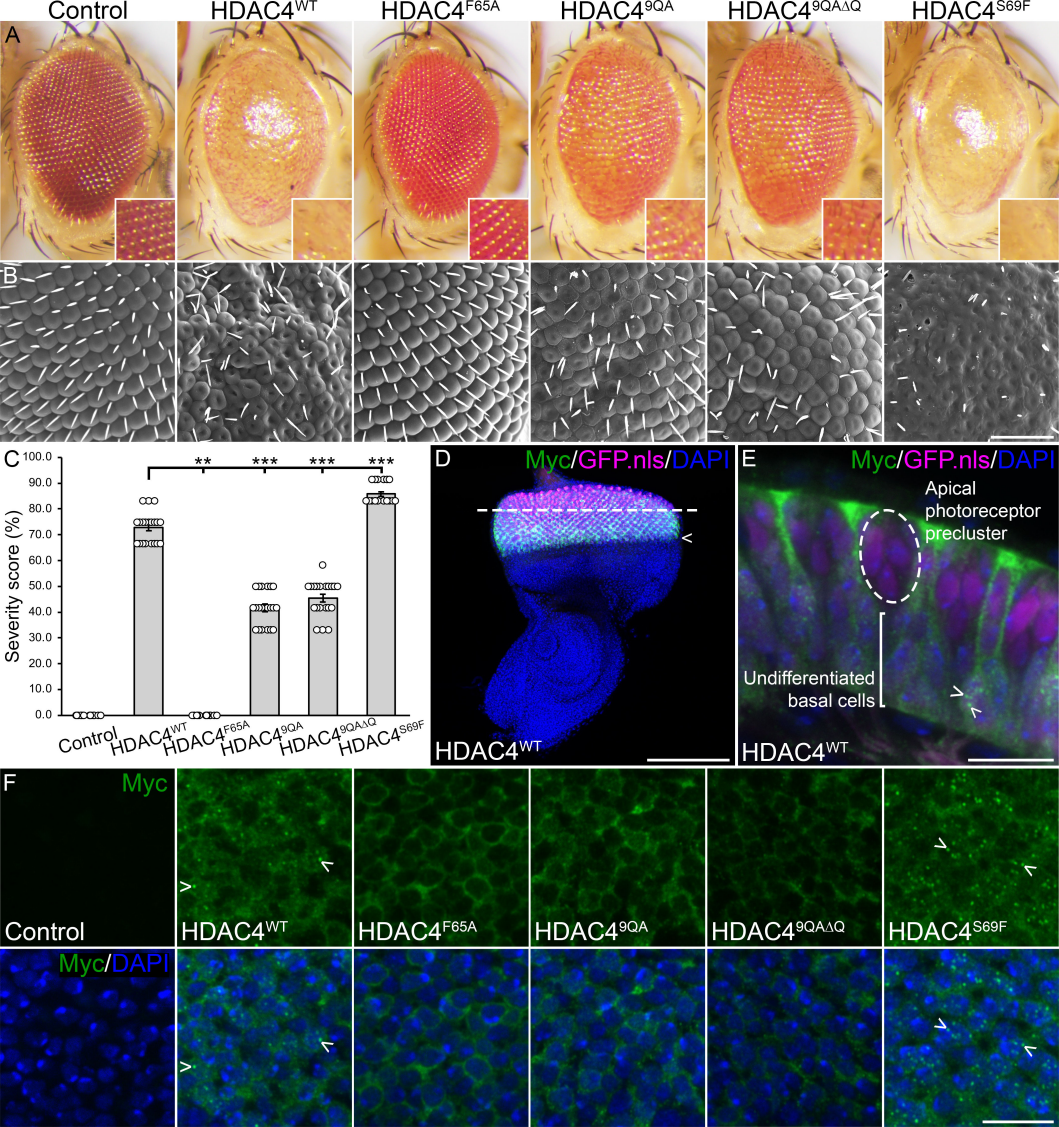
Oligomerization mutants alter HDAC4-overexpression-induced defects in eye development. (A) Stereomicrographs (110× magnification) and (B) scanning electron micrographs of adult *Drosophila* eyes of flies raised at 25°C. Flies carry one copy of *GMR-GAL4* and two copies of the *HDAC4-Myc* wild-type or mutant transgene (*GMR-GAL4/+; HDAC4/HDAC4*). The control is *GMR-GAL4/+; +*. Scale bar = 50 µm. (C) Quantification of eye phenotype severity. HDAC4^F65A^, HDAC4^9QA^ and HDAC4^9QA∆Q^ mutants were significantly reduced, and HDAC4^S69F^ was significantly increased in severity compared with HDAC4^WT^. ANOVA, *F*_(5,112)_ = 1642.19, *p* < 0.001, *post hoc* Tukey’s HSD, ***p < 0.001. Error bars indicate SEM. (D) Maximum projection of a third instar larval eye disc expressing HDAC4^WT^ (green) and GFP.nls (magenta) under the control of *GMR-GAL4*. Flies were raised at 25°C. Expression of both HDAC4^WT^ and GFP.nls was observed posterior to the morphogenetic furrow (arrowhead). DAPI (blue) highlights nuclei across the whole disc. The dashed line indicates the location of the cross section depicted in (E). Scale bar = 100 µm. (E) Cross section of the larval eye disc demonstrating that HDAC4^WT^ localizes to both the apical and basal cells of the disc, but only forms small nuclear condensates (arrowheads) in undifferentiated basal cells. A photoreceptor precluster that forms a single ommatidium is circled in the apical surface, where HDAC4 condensates were not observed. Scale bar = 10 µm. (F) Single optical sections (0.5 µm) of basal undifferentiated cells of the larval eye disc expressing HDAC4^WT^ or HDAC4^Mutant^-Myc (green), co-labelled with DAPI (blue). Scale bar = 10 µm.

Overexpression of HDAC4 is also associated with impaired eye development in *Drosophila* [35,42,43]. Development of the *Drosophila* eye initiates in early larval development and this process has been extensively characterized [58–60]. To investigate whether the defects in eye development similarly correlate with HDAC4 condensation, we used the *glass multimer reporter* driver (*GMR-GAL4*) to express HDAC4 transgenes in post-mitotic photoreceptors [61]. Expression of HDAC4^WT^ resulted in significant morphological abnormalities including fusion, and disorganization of ommatidia, loss of pigmentation, and the absence of bristles (figure 5A,B), consistent with previous findings [35,42,43]. Similar disruption was also observed for HDAC4^WT^-HA but not for HDAC4^M1-L285^-HA, indicating that the phenotype is dependent on the full-length protein, and independent of the epitope tag (electronic supplementary material, figure S4). HDAC4^F65A^ had no detectable effect on eye morphology (figure 5A–C), suggesting that disruption of oligomerization via this interface abrogates HDAC4’s ability to impair eye development. By contrast, HDAC4^9QA^ and HDAC4^9QA∆Q^ produced milder but still detectable eye defects, pointing to a more complex role for these mutants. Expression of HDAC4^S69F^ exacerbated the defects with almost complete fusion of all ommatidia and formation of cavities. To directly correlate changes in severity of the eye phenotype with condensation of HDAC4, immunohistochemistry was performed on the third instar larval eye disc (figure 5D), which represents the developmental window during which the adult eye is patterned and formed. As previously reported [35], HDAC4^WT^ formed prominent nuclear condensates in the undifferentiated basal cells of the disc (figure 5E,F). HDAC4^S69F^, which exacerbated eye defects, showed increased condensation. By contrast, HDAC4^F65A^, HDAC4^9QA^ and HDAC4^9QA∆Q^ showed markedly fewer and smaller nuclear condensates, often appearing as cytoplasmic haloes rather than concentrated nuclear foci (figure 5F).

Collectively these data further support a model in which HDAC4 condensation, mediated by self-oligomerization through the N-terminal α-helix, generally correlates with developmental defects in both the eye and mushroom body. However, this is not strictly linear—the differential phenotypes observed for HDAC4^F65A^ and HDAC4^9QA^ in the eye, despite both showing reduced condensation, highlight that additional factors such as protein stability, nuclear trafficking, or altered interactions with endogenous partners may modulate function in a tissue-specific manner. Thus, while nuclear condensation appears to be a key feature associated with HDAC4-induced pathology, the biological consequences of disrupting this process can differ depending on the nature of the mutation and the cellular context.

### 2.5. Both HDAC4 oligomerization and MEF2 regulate nuclear entry

HDAC4 undergoes nucleocytoplasmic shuttling in response to stimuli [3,24–27], with subcellular distribution differing across cell types and species [9,42]. Despite intrinsic nuclear import and export signals, nuclear import of HDAC4 is primarily mediated through MEF2 binding, with point mutations that abrogate MEF2 binding resulting in cytoplasmic retention [14]. Previous studies showed that introducing the Phe93Asp mutation into hsHDAC4 (analogous to DmHDAC4^F65A^) impaired MEF2-dependent transcriptional repression [36,44] suggesting that HDAC4 oligomerization may be necessary for MEF2 binding and proper subcellular localization.

**Figure 6.**
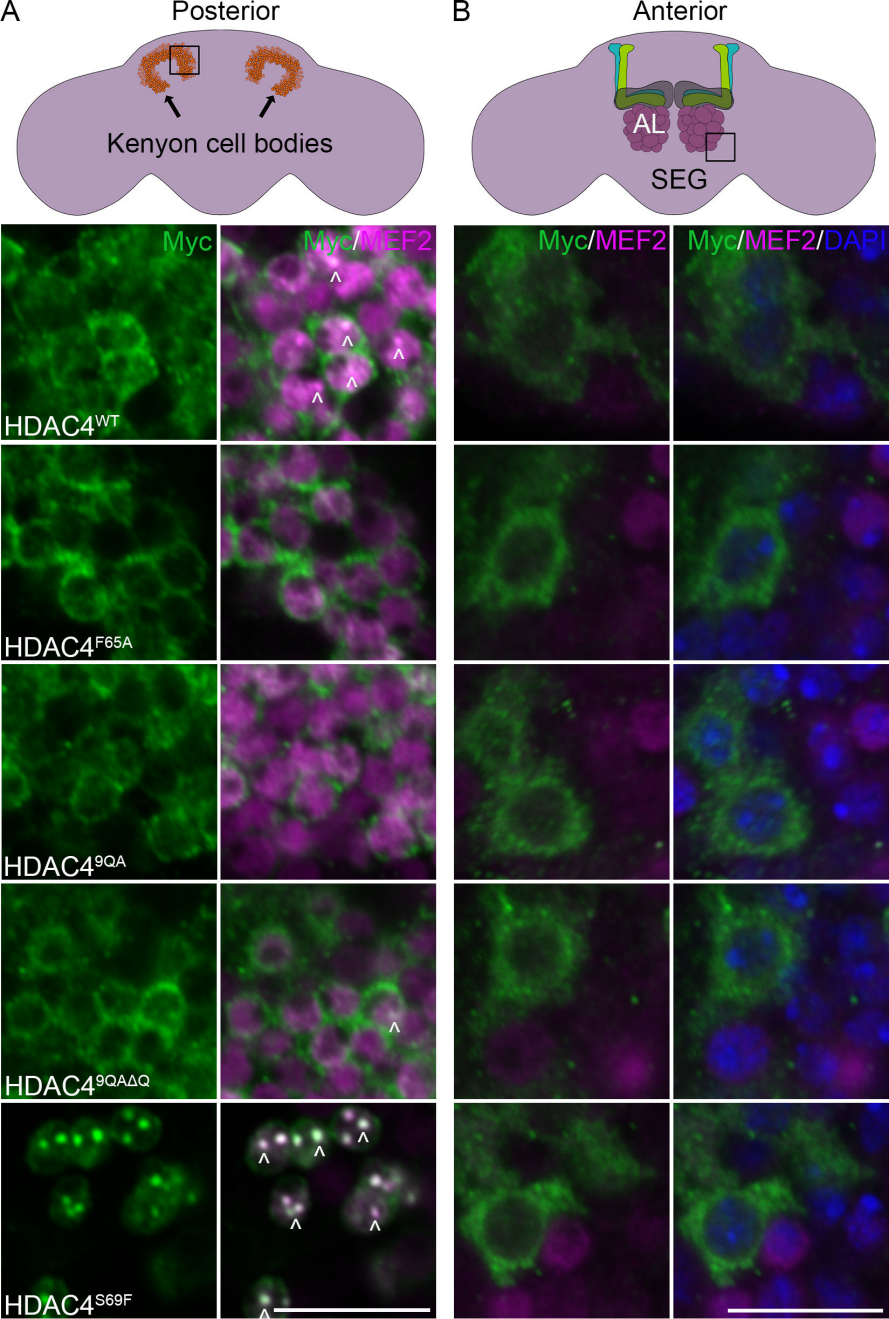
Expression and subcellular localization of HDAC4 oligomerization mutants in the adult brain. All genotypes were generated by crossing *elav-GAL4* females to males carrying the indicated *UAS-HDAC4-Myc* transgene. (A) Anti-Myc (green) and anti-MEF2 (magenta) immunohistochemistry was performed on whole brains. The schematic shows the Kenyon cell bodies viewed from the posterior, with the boxed region imaged via single optical 0.5 µm sections through the calyx and Kenyon cell layer. Arrowheads point to nuclear condensates of HDAC4. Scale bar = 10 µm. (B) Anti-Myc (green) and anti-MEF2 (magenta) immunohistochemistry and counterstaining with DAPI (blue) was performed on whole brains. The schematic shows the suboesophageal ganglion (SEG) and antennal lobe (AL) viewed from the anterior with the boxed region imaged via single optical 0.5 µm sections of the cells surrounding the AL and SEG. Scale bar = 10 µm.

To explore this, we examined HDAC4 distribution in the *Drosophila* adult brain using *elav-GAL4*. Within Kenyon cells, HDAC4^WT^-Myc localized to the nucleus and cytoplasm with relatively uniform distribution and condensates were visible (figure 6A). Strikingly, HDAC4^F65A^, HDAC4^9QA^ and HDAC4^9QAΔQ^ displayed decreased nuclear localization and formed perinuclear haloes, whereas HDAC4^S96F^ fully localized to the nucleus where it formed prominent condensates.

By contrast, in cells surrounding the antennal lobes and the suboesophageal ganglion, HDAC4^WT^ was predominantly cytoplasmic, forming perinuclear haloes (figure 6B) and this distribution was unchanged for any of the mutants. Notably, these cells express a much lower level of MEF2 than Kenyon cells and MEF2 did not localize to cells in which HDAC4 was expressed. Together these findings indicate that HDAC4 subcellular distribution is cell type-specific, and its nuclear localization is closely associated with MEF2 expression, supporting a model in which oligomerization facilitates nuclear entry via MEF2 binding.

### 2.6. Both HDAC4 oligomerization and MEF2 binding contribute to HDAC4 condensation

To specifically examine the role of oligomerization in condensate formation without the confounding effects of nuclear import, we generated HDAC4 3SA mutants by substituting serine residues at 14-3-3 binding sites with alanine (S239A, S573A, S748A), thereby blocking nuclear export [14,21,35]. These constructs allowed us to compare condensate formation across mutants with equivalent nuclear retention. Specifically, we introduced the F65A and 9QA mutations into the 3SA background to generate HDAC4^3SA-F65A^ and HDAC4^3SA-9QA^ , and also generated an HDAC4^3SA-F65A-ΔMEF2^ mutant to examine the contribution of MEF2 binding. Expression of all variants was confirmed via western blot (electronic supplementary material, figure S5A,B) and fractionation confirmed that the 3SA mutation increased their nuclear retention (electronic supplementary material, figure S5C–E), enabling unbiased comparison of nuclear condensation.

**Figure 7.**
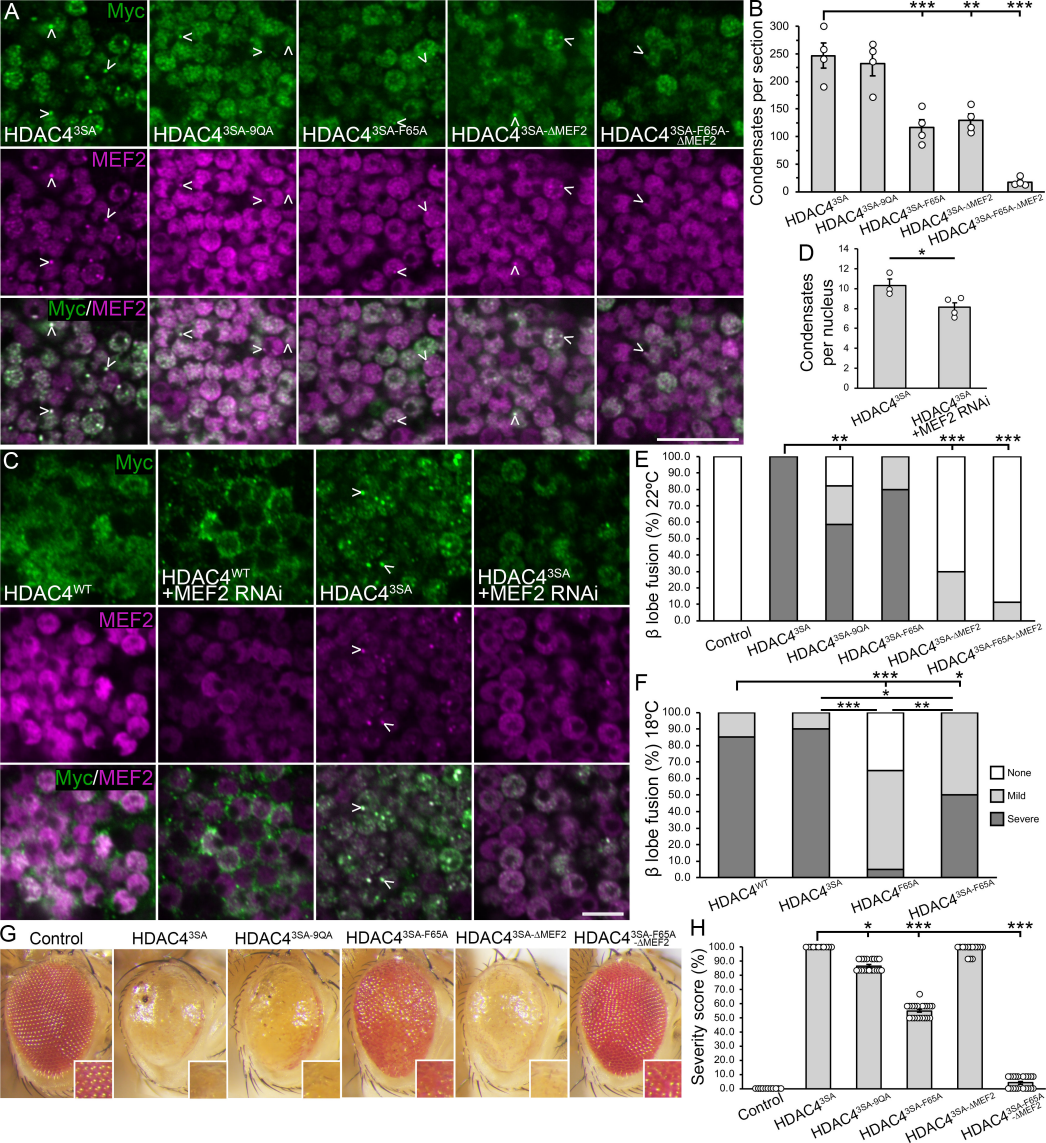
Functional analysis of nuclear HDAC4 oligomerization mutants and the role of MEF2. (A) Immunostaining of whole brains with anti-Myc (green) and anti-MEF2 (magenta). The indicated transgenes were driven with *OK107-GAL4* and induced in adulthood with GAL80ts (30°C, 72 h). Representative 0.5 µm sections through the Kenyon cell layer are shown. Arrowheads point to nuclear condensates of HDAC4. Scale bar = 10 µm. (B) Quantification of nuclear condensates shown in (A). ANOVA, *F*_(4,15)_ = 32.65, *p* < 0.0001; *post hoc* Tukey’s HSD, **p < 0.01, ***p < 0.001. Error bars indicate SEM. (C) Immunostaining of whole brains with anti-Myc (green) and anti-MEF2 (magenta). Flies were raised as in (A). Representative 0.5 µm sections through the Kenyon cell layer are shown. Arrowheads point to nuclear condensates of HDAC4. Scale bar = 10 µm. (D) Quantification of HDAC4^3SA^ condensates shown in (C). Condensates were counted and averaged for *n* ≥ 43 nuclei per section for *n* ≥ 3 brains per genotype. Unpaired *t*‐test, HDAC4^3SA^ versus HDAC4^3SA^ + MEF2 RNAi *t*_(5)_ = 2.759, *p* = 0.0399. Error bars indicate SEM. (E,F) Quantification of β lobe fusion resulting from HDAC4 transgene expression. (E) Flies were raised at 22°C. HDAC4^3SA-9QA^, HDAC4^3SA-∆MEF2^ and HDAC4^3SA-F65A-∆MEF2^ mutants all displayed a significant reduction in severe β lobe fusion compared with HDAC4^3SA^, *p* = 0.0019, *p* < 0.0001 and *p* < 0.0001, respectively. *p < 0.05, **p < 0.01, ***p < 0.001, Fisher’s exact test. Error bars indicate SEM. (F) Flies were raised at 18°C. HDAC4^3SA-F65A^ mutants displayed a significant reduction in severe β lobe fusion compared with HDAC4^3SA^, *p* = 0.0138. *p < 0.05, **p < 0.01, ***p < 0.001, Fisher’s exact test. Error bars indicate SEM. (G) Stereomicrographs (110× magnification) of adult *Drosophila* eyes of flies raised at 25°C. Flies carry one copy of *GMR-GAL4* and two copies of the *HDAC4^Mutant^-Myc* transgene (*GMR-GAL4/+; HDAC4/HDAC4*). The control is *GMR-GAL4/+; +*. (H) Quantification of eye phenotype severity. Significant differences between HDAC4^3SA^ and HDAC4^3SA-Mutant^ were observed. Kruskal–Wallis test, *H*_(5)_ = 106.92, *p* < 0.001, *post hoc* Dunn’s test, *p < 0.05, ***p < 0.001. HDAC4^3SA^ versus HDAC4^3SA-9QA^, *p* = 0.01561; versus HDAC4^3SA-F65A^, *p* < 0.001; versus HDAC4^3SA-∆MEF2^, *p* = 0.7861; versus HDAC4^3SA-F65A-∆MEF2^ *p* < 0.001. HDAC4^3SA-F65A^ versus HDAC4^3SA-F65A-∆MEF2^ *p* = 0.01387. Control versus HDAC4^3SA-F65A-∆MEF2^ *p* = 0.3387. Error bars indicate SEM.

Condensate analysis revealed that HDAC4^3SA-F65A^ formed fewer condensates than HDAC4^3SA^ (figure 7A,B), indicating that oligomerization contributes to condensate formation. Disrupting both oligomerization and MEF2 binding via expression of HDAC4^3SA-F65A-ΔMEF2^ had an additive effect, resulting in almost complete abolishment of condensates. To confirm the role of MEF2 in condensate stabilization, we knocked down MEF2 via RNAi. This resulted in increased cytoplasmic localization of HDAC4^WT^ (figure 7C), consistent with MEF2-dependent nuclear import. By contrast, HDAC4^3SA^, which is restricted to the nucleus due to blocked export, remained nuclear but formed fewer condensates upon MEF2 knockdown (figure 7C,D). These results indicate that MEF2 also contributes to the stabilization of HDAC4 condensates in the nucleus.

### 2.7. Oligomerization and MEF2 binding drive HDAC4-induced neurodevelopmental defects

We next examined how these mutations affected mushroom body development. Expression of HDAC4^3SA^ caused severe β lobe fusion in 100% of brains, while HDAC4^3SA-9QA^ reduced this to 59% (table 3, figure 7E). HDAC4^3SA-F65A^ expression decreased severe β lobe fusion to 80%, which was not significantly different from HDAC4^3SA^. Strikingly, HDAC4^3SA-ΔMEF2^ and HDAC4^3SA-F65A-ΔMEF2^ mutations eliminated severe fusion, and total fusion was reduced to 30% and 11% respectively. Since HDAC4^3SA^ expression already caused maximal β lobe fusion, we lowered the temperature to reduce expression. Under these conditions, severe fusion was observed in 90% of HDAC4^3SA^ brains, but was reduced to 50% in HDAC4^3SA-F65A^ brains (figure 7F). Together these data demonstrate that mutations impairing HDAC4 oligomerization reduce β lobe fusion, independent of subcellular distribution.

**Table 3.** Nuclear-restricted HDAC4 oligomerization mutants reduce HDAC4-overexpression-induced defects in mushroom body development. All genotypes were generated by crossing *elav-GAL4* females to males carrying each *UAS-HDAC4-Myc* transgene and to the *w*(*CS10*) control. The percentage of brains displaying each phenotype was calculated from the total number of brains analysed for each genotype (*n*). At 22°C overexpression of HDAC4^3SA^ resulted in a significant number of brains with β lobe fusion compared with control (*p* < 0.0001, Fisher’s exact test). HDAC4^3SA-9QA^, HDAC4^3SA-∆MEF2^ and HDAC4^3SA-F65A-∆MEF2^ mutants each significantly reduced severe β lobe fusion compared with HDAC4^3SA^ (*p* = 0.0019, *p* < 0.0001 and *p* < 0.0001, respectively). At 18°C severe β lobe fusion was significantly reduced for HDAC4^3SA-F65A^ compared with HDAC4^3SA^ (*p* = 0.0138). Severe β lobe fusion was reduced for HDAC4^F65A^ compared with HDAC4^WT^ (*p* < 0.0001). Percentages may not sum to 100 due to rounding.

	22°C						18°C				
(% of brains)	control	HDAC4 3SA	HDAC4 3SA-9QA	HDAC4 3SA-F65A	HDAC4 3SA-∆MEF2	HDAC4 3SA-F65A-∆MEF2	control	HDAC4 WT	HDAC4 3SA	HDAC4 F65A	HDAC4 3SA-F65A
α lobe thinning	0	50	27	35	5	0	0	10	40	0	10
α lobe missing	0	6	24	0	0	0.0	5	0	5	0	0
β lobe thinning	0	13	9	5	0	6	0	0	10	0	15
β lobe missing	0	13	0	5	5	11	0	0	5	10	5
β lobe fusion	0	100	82	100	30	11	20	100	100	65	100
mild	0	0	24	20	30	11	20	15	10	60	50
severe	0	100	59	80	0	0	0	85	90	5	50
guidance defect	0	50	32	45	10	11	5	0	50	15	40
no defects	100	0	6	0	60	78	75	0	0	25	0
*n*	20	16	34	20	20	18	20	20	20	20	20

In the eye, expression of HDAC4^3SA^ caused severe developmental disruption, including complete fusion of ommatidia, loss of pigmentation, loss of bristles and necrosis (figure 7G,H). Expression of HDAC4^3SA-9QA^ slightly reduced the severity with fewer necrotic spots, though fusion and loss of pigmentation remained. HDAC4^3SA-F65A^ provided clearer improvement, with some restoration of pigmentation and ommatidial structure. Notably, HDAC4^3SA-F65A-∆MEF2^ almost fully rescued the phenotype, producing eyes that appeared largely wild-type. By contrast, HDAC4^3SA-ΔMEF2^ showed only minor improvement, suggesting that oligomerization is essential for HDAC4-induced eye disruption. Since we previously illustrated that MEF2 binding is not required by HDAC4 to disrupt eye development, and detected no endogenous expression of MEF2 in the developing eye [35], these findings also suggest that the MEF2 binding site may facilitate interactions with an unidentified factor required for HDAC4-induced neurodevelopmental disruption in this tissue. Thus, both HDAC4 oligomerization and the MEF2 binding site are required for HDAC4-induced developmental disruption in the eye.

## 3. Discussion

Here we demonstrate that oligomerization of HDAC4 is essential for nuclear condensation and contributes to overexpression-induced neurodevelopmental defects in *Drosophila*. Using mutants that disrupt oligomerization and/or MEF2 binding, we dissected the contributions of these domains to condensate formation and neurotoxicity. Mutations in the N-terminal region that impaired oligomerization (HDAC4^F65A^ and HDAC4^9QA^) reduced condensate number *in vivo*, and this correlated with reduced severity of HDAC4-induced phenotypes in the mushroom body and adult eye, although this was not a linear relationship, as further discussed below. The relationship between nuclear condensates and phenotype severity was also somewhat confounded by the fact that these mutations also reduced the nuclear abundance of HDAC4. To resolve this, we enforced nuclear localization using a 3SA mutant background that disrupts 14-3-3-mediated export. Even when confined to the nucleus, HDAC4^3SA-F65A^ formed fewer condensates, confirming a direct role for oligomerization in condensate formation. Importantly, additional disruption of MEF2 binding further destabilized condensates and improved developmental outcomes, especially in the eye. These findings underscore a cooperative role for oligomerization and MEF2 binding in HDAC4 nuclear accumulation and subsequent condensate formation. Together, these findings establish HDAC4 oligomerization and MEF2 binding as key drivers of HDAC4-induced neurotoxicity, linking nuclear condensates to neurodevelopmental pathology.

HDAC4 is observed in small granular or punctate nuclear and cytoplasmic foci in neurons of both healthy mouse and *Drosophila* brains [9,35], suggesting that condensate formation may be a feature of its normal function. We hypothesize that small, dynamic HDAC4 condensates act as regulatory sites, potentially through interaction with transcription factors such as MEF2. Upon increased HDAC4 nuclear abundance, whether by overexpression, impaired import or unknown disease mechanisms, these puncta may increase in size and number, potentially reflecting fusion or coalescence events, and we hypothesize that this may induce neuronal dysfunction. While we observed such events for GFP-tagged HDAC4 under overexpression conditions, whether this occurs for endogenous HDAC4 remains to be investigated.

We determined that the mechanism of HDAC4 oligomerization is conserved between *Drosophila* and humans. In *Drosophila*, mutations that disrupt either the hydrophobic core or polar interaction networks in the N-terminus impair oligomerization *in vitro* and reduce condensation of full-length HDAC4 *in vivo*. These structural features are conserved across class IIa HDACs [36], suggesting oligomerization is critical to their function. Supporting this, HDAC4 oligomerization is required for repression of MEF2-dependent transcription *in vitro* [36,44]. *In vivo*, oligomerization-disrupting mutants led to increased cytoplasmic distribution of HDAC4 in Kenyon cells. This is likely due to impaired MEF2 binding, which is known to promote nuclear import [14,23] and increase condensation [35].

We also explored the role of glutamine-rich regions, which are frequently implicated in protein aggregation [62]. Mutating the glutamine residues implicated in oligomer stability (HDAC4^9QA^) reduced protein stability and *in vivo* condensation, and purified HDAC4^N-9QA^ was unstable *in vitro*. In cross-linking assays, the HDAC4^9QA^ mutant failed to form defined dimers or higher-order oligomers, instead producing a smeared profile suggestive of oligomerization of degradation products, or aberrant aggregation. This instability limited our ability to assess the precise contribution of this mutant to HDAC4 function. The decreased level of condensation observed *in vivo* was at least partially attributable to reduced protein levels rather than impaired oligomerization alone. Phenotypically, HDAC4^9QA^ induced milder mushroom body defects than HDAC4^WT^, consistent with its lower expression and inability to oligomerize. Unexpectedly, HDAC4^9QA^ produced an intermediate phenotype in the eye, while HDAC4^F65A^ had no phenotype. This suggests that the 9QA mutation, in addition to blocking oligomerization, may alter HDAC4 structure or interaction specificity in a way that affects eye development.

This structural complexity highlights the value of HDAC4^F65A^ as a more informative model for dissecting the role of oligomerization. Unlike HDAC4^9QA^, HDAC4^F65A^ maintained wild-type protein levels and preserved its α-helical conformation, yet exhibited reduced oligomerization and condensation. Correspondingly, HDAC4^F65A^-induced phenotypes were milder than HDAC4^WT^, particularly in the mushroom body, and absent in the eye. Thus HDAC4^F65A^ provided a cleaner experimental tool to examine the specific effects of reduced oligomerization without confounding structural instability.

Importantly, disruption of the MEF2-binding domain or knockdown of MEF2 reduced nuclear condensation independently of HDAC4 self-oligomerization and subcellular distribution, indicating that MEF2 not only facilitates nuclear localization but also stabilizes HDAC4 condensates. These findings raise the possibility that altered MEF2 expression could impair HDAC4 nucleocytoplasmic dynamics and contribute to its nuclear accumulation and condensation in neuronal disease. Further supporting a functional relationship between oligomerization and MEF2 binding, it has been shown that binding of MEF2’s HDAC-interacting domain to the N-terminus of human HDAC4 promotes dimerization and chromatin looping through the same hydrophobic core that mediates tetramerization [36,44]. This reinforces the idea that HDAC4 oligomerization underlies its chromatin regulatory functions.

Consistent with characteristics of LLPS, HDAC4 condensates were dose-dependent and dynamic. We show that HDAC4 nuclear condensation correlates with increased nuclear abundance, which has been observed in disease [28–30,33]. HDAC4 condensates are not β-sheet insoluble inclusions, but instead are dynamic and display features of LLPS in that their formation is both concentration- and interaction-dependent. Phase separation involves a nucleation event that seeds condensate formation [63], often involving a scaffold protein that recruits clients. HDAC4 oligomerization may constitute this nucleation trigger, with MEF2 acting as a client that further stabilizes condensation. Another key feature of LLPS is valency, which describes how the scaffold protein has many binding regions and motifs, including intrinsically disordered regions (IDRs) [40]. Indeed regions outside of the N-terminus of human HDAC4 are predicted to be intrinsically disordered [39] and are essential for *Drosophila* HDAC4 condensation, supporting a conserved role in phase separation.

Our data also point to a dominant negative effect of HDAC4 condensates. We previously demonstrated that endogenous HDAC4 is sequestered in condensates that result from GAL4-induced expression of transgene HDAC4 [35]. Here we show that oligomerization-reduced mutants still formed nuclear condensates when localized to the nucleus, likely supported by binding endogenous HDAC4. This could explain how heterozygous disease-associated mutations that disrupt 14-3-3 binding, which are predicted to promote nuclear retention [6], could contribute to pathology by driving condensation with wild-type HDAC4, potentially disrupting its normal function. These mutations have further significance in light of the recent finding that 14-3-3 binding to HDAC4 destabilizes condensate formation [39]. Further research is required to understand the relationship between the concentration of endogenous HDAC4 and condensate dynamics.

Tissue specific differences in HDAC4 function were evident. Although the MEF2 binding site is largely dispensable for HDAC4-induced disruption of eye development [35], its loss enhanced the phenotypic rescue conferred by HDAC4^3SA-F65A^. Despite the presence of MEF2 in the optic lobes of the brain [64], we have not been able to detect endogenous expression within developing photoreceptor nuclei [35], suggesting the MEF2 binding site of HDAC4 may mediate interactions with other critical factors. One possibility is serum response factor, which shares an overlapping binding site with MEF2 [14,65], and is implicated in the *Drosophila* eye development [66]. These findings highlight the need to consider cell-type-specific binding partners in understanding HDAC4 condensation and toxicity.

## 4. Conclusion

HDAC4 condensation is a dynamic process, dependent on oligomerization, dose, subcellular distribution and protein–protein interactions. We demonstrate a correlation between nuclear condensation of HDAC4 and neurodevelopmental defects *in vivo*, providing evidence that HDAC4 condensates contribute to neuronal dysfunction. MEF2 is a key partner that promotes both nuclear import and condensate stability, but additional interacting proteins likely contribute in a tissue-specific manner. These findings clarify the molecular basis of HDAC4 condensation, and highlight its potential as a therapeutic target for disorders in which HDAC4 regulation is disrupted.

## 5. Methods

### 5.1. Fly strains

All flies were maintained on standard medium at 22°C with a 12 h light–dark cycle unless otherwise indicated. *P{w[+mW.hs] = GawB}elav[c155]* (*elav-GAL4*, BDSC 458), *w[∗]; P{w[+mW.hs] = GawB}OK107 ey[OK107]/In(4)ci[D], ci[D] pan[ciD] sv[spa-pol]* (*OK107-GAL4*, BDSC 854), *w[∗ ]; P{w[+mC] = GAL4-ninaE.GMR}12* (*GMR-GAL4*, BDSC 1104), *w[1118]; P{w[+mC] = UAS-GFP.nls}14* (*UAS-GFP.nls*, BDSC 4775) and P{w[+mC] = UAS-APP.Abeta42.B}m26a (*UAS-Ab42*, BDSC 33770) were obtained from the Bloomington *Drosophila* Stock Center. *w∗; P{w+mC = tubP-GAL80ts}10; TM2/TM6B, Tb1* (*tubP-GAL80ts*) and *w*(*CS10*) strains were provided by R. Davis (Scripps Research Institute, Jupiter, FL). P{GD5039}v15550 (UAS-*MEF2-RNAi*, VDRC 15550) was obtained from the Vienna *Drosophila* Resource Center. *GFP-HDAC4^WT^* was previously generated [42]. Homozygous lines carrying *OK107-GAL4*, *tubP-GAL80ts* and both *GFP-HDAC4^WT^* and *HDAC4^Mutant^-Myc* were generated by standard genetic crosses. The open reading frame of wild-type *DmHDAC4* was previously synthesized (nucleotides 461−4216, NCBI NM_132640) with a C-terminal 6× Myc tag [42], *HDAC4^WT^-Myc*, and mutagenesis performed by GenScript (New Jersey, USA) to obtain *HDAC4^F65A^*, *HDAC4^9QA^*, *HDAC4^9QA∆Q^*, *HDAC4^S69F^*, *HDAC4^3SA-9QA^*, *HDAC4^3SA-F65A^*, *HDAC4^3SA-F65A-∆MEF2^*, *as well as HDAC4^3SA^ and HDAC4^3SA-∆MEF2^* as previously described [42]. Replacement of the *HDAC4^WT^* 6× Myc tag with a 3× HA tag was carried out by Genscript. *HDAC4^M1-L285^-HA* (nucleotides 461–1315, NCBI NM_132640), with the Ser239Ala mutation was synthesized with a C-terminal 3× HA tag. *HDAC4* constructs were cloned into the pUASTattB vector for germline transformation of *Drosophila* (GenetiVision, Houston, TX) using the P2 docking site at (3L)68A4. All strains were outcrossed for a minimum of five generations into the *w*(*CS10*) genetic background.

### 5.2. Protein expression, purification and biochemical analyses

Protein sequence alignment of *Drosophila* (nucleotides 461–4216, NCBI NM_132640, translated) and human (NCBI NP_006028) HDAC4 was performed using EMBOSS Water pairwise sequence alignment (Madeira, Madhusoodanan [67], https://www.ebi.ac.uk/jdispatcher/psa/emboss_water, RRID:SCR_025141). Residues Ala62-Gln153 of hsHDAC4 were previously examined for tetramer formation [36] and the corresponding region of DmHDAC4 was determined as Pro37-Gln143. This region was codon optimized for *E. coli* and synthesized by GenScript to create HDAC4^N-WT^. This was followed by mutagenesis to create HDAC4^N-F65A^, HDAC4^N-9QA^ and HDAC4^N-S69F^. HDAC4^N^ constructs were cloned into the pET-15B expression vector in frame with the N-terminal 6× His tag. HDAC4^N^ were transformed into BL21(DE3) *E. coli* for recombinant protein expression. Cells were grown at 30°C in 2× YT culture medium containing ampicillin (100 µg ml^−1^) until OD_550_ reached approximately 0.6, at which time expression was induced with 0.5 mM IPTG and cells were grown at 20°C overnight (approx. 14 h, HDAC4^WT^ and HDAC4^S69F^, 1 l total culture volume) or at 25°C for 3 h (HDAC4^F65A^ and HDAC4^9QA^, 10 l total culture volume). Cells were harvested by centrifugation at 5000 × *g* for 10 min and pellets stored at −80°C. Pellets were thawed and resuspended in 20 ml (per litre of culture) lysis buffer (30 mM HEPES, pH 7.5, 300 mM NaCl, 10 µg ml^−1^ Pepstatin A, 1× cOmplete EDTA-free protease inhibitors (Roche)) before lysis via Cell Disruptor (Constant Systems) at 10 kpsi and sonication for 2 × 30 s pulses at 20 W. The lysate was clarified by centrifugation at 20 000 × *g* for 30 min before filter sterilization. The supernatant was subjected to nickel-affinity chromatography using Profinity IMAC Resin (BioRad) in IMAC buffer (30 mM HEPES, pH 7.5, 300 mM NaCl) with a gradient elution from 30 mM to 500 mM imidazole. A_215_ and A_230_ were examined to determine the fractions in which HDAC4^N^ eluted, and these were also run on SDS-PAGE to confirm HDAC4^N^ presence given the low number of tyrosine and tryptophan residues. Pooled fractions containing HDAC4^N^ were subjected to size exclusion chromatography on a Superdex 75 10/300 GL column (Cytiva) in SEC buffer (30 mM HEPES, pH 7.6, 150 mM NaCl), and fractions containing HDAC4^N^ were pooled and concentrated to approximately 0.5−2 mg ml^−1^ before snap freezing and storing at −80°C.

Crosslinking was performed as per Guo *et al*. [36]. Briefly, 0.5 mg ml^−1^ HDAC4^N^ diluted in SEC buffer (20 µl) was incubated with 2 µl disuccinimidyl suberate (DSS, Thermo Fisher Scientific) dissolved in DMSO for 30 min at room temperature. The reaction was quenched for 15 min with 1 µl of 1 M Tris (pH 7.5) before 5 µl was prepared for SDS-PAGE followed by colloidal Coomassie staining. Quantification was performed using the Gel Analyser plugin within ImageJ [68] (https://imagej.net/, RRID:SCR_003070). Proportional intensity was calculated and data transformed using centred log-ratio before performing a one-way ANOVA with *post hoc* Tukey’s HSD test with significance set at *α* = 0.05.

Circular dichroism was performed on HDAC4^N^ prepared in SEC buffer at 0.5 mg ml^−1^ in a 0.1 mm quartz cuvette. Spectra were recorded using a Chirascan spectrophotometer (Applied Photophysics). For each sample spectra were collected between 180 and 260 nm in 1 nm steps and averaged, and data were baseline corrected for the sample buffer.

### 5.3. SDS-PAGE and western blotting

Whole cell lysates were produced by homogenizing snap-frozen *Drosophila* heads in either IP buffer (50 mM sodium chloride, 30 mM sodium pyrophosphate, 50 mM sodium fluoride, 10% glycerol, 0.5% Triton X-100, 0.5 mM PMSF, 25 mM Tris, pH 7.05, 1× protease inhibitors (cOmplete EDTA free Protease Inhibitor Cocktail (Roche)) or RIPA buffer (150 mM sodium chloride, 0.1% Triton X-100, 0.5% sodium deoxycholate, 0.1% SDS, 50 mM Tris pH 8.0, 1× protease inhibitors) and collecting the supernatant following centrifugation at 12 000 × *g* for 2 min. Nuclear and cytoplasmic lysates were prepared using the NE-PER nuclear and cytoplasmic extraction reagents (Thermo Fisher Scientific) using a protocol modified from Maitra *et al*. [69]. Heads (*n* = 50) were homogenized in 100 µl of cytoplasmic buffer 1 (CB1) supplemented with protease inhibitors and incubated on ice for 10 mins before adding 5.5 µl of cytoplasmic buffer 2 (CB2) and centrifugation at 10 000 × *g* for 5 min. The supernatant was retained as the cytoplasmic fraction, and the pellet washed twice by resuspending in 50 µl of CB1, incubating for 10 min, then adding 2.75 µl of CB2, centrifuging at 10 000 × *g* for 5 min and discarding the supernatant. The pellet was resuspended in 50 µl of nuclear extraction reagent with protease inhibitors, incubated on ice for 40 min and centrifuged at 10 000 × *g* for 10 min before collecting the supernatant as the nuclear fraction. Total protein was quantified using a Pierce BCA Protein Assay kit (Thermo Fisher Scientific). Whole cell lysate (30 µg) or subcellular fractions (20 µg) were denatured in 1× sample buffer (2% SDS, 5% 2-mercaptoethanol, 10% glycerol, 0.01% bromophenol blue, 60 mM Tris HCl, pH 6.8) at 90°C for 5 min. Samples were loaded onto 4–20% or 10% Mini-PROTEAN TGX Precast Protein gels (BioRad) and electrophoresed at 200 V. Protein was blotted onto a nitrocellulose membrane for 1 h at 4°C before blocking in 5% skim milk powder in TBST (20 mM Tris, 150 mM NaCl, pH 7.6, 0.1% Tween-20) for 1 h at room temperature. Following washing in TBST membranes were incubated overnight at 4°C in primary antibody in 1% skim milk powder/TBST, and 1 h at room temperature in secondary antibody. Antibodies used were rabbit anti-Myc (ab9106, Abcam, 1:1000, Antibody Registry Identifier RRID:AB_307014), rabbit anti-GFP (ab290, Abcam, 1:4000, RRID:AB_303395), rat anti-HA (Roche, clone 3F10, 1:1000, RRID:AB_2687407), mouse anti-α-tubulin (12G10 clone, Developmental Studies Hybridoma Bank (DSHB), 1:500, RRID:AB_1157911), mouse anti-lamin(Dm0) (ADL67.10 clone, DSHB, RRID:AB_528336), sheep anti-mouse-HRP (Sigma Aldrich NA931VS, 1:20 000), donkey anti-rabbit-HRP (Sigma Aldrich NA934VS, 1:40 000), goat anti-rat-HRP (Abcam ab97057, 1:10 000, RRID:AB_10680316). Amersham ECL Prime detection reagent (GE Life Sciences) was used as per manufacturer’s instructions, and chemiluminescence detected using the Azure Biosystems C600 imaging system. Quantification of western blots was performed using the Gel Analyser plugin within ImageJ [68] (https://imagej.net/, RRID:SCR_003070). Whole cell and cytoplasmic lysate signal were normalized to tubulin, and nuclear lysate to lamin. For blots of whole cell lysates, the fold-change of the normalized signal was calculated relative to HDAC4^WT^. For blots from subcellular fractionation, the nuclear to cytoplasmic ratio was calculated for each sample and normalized to HDAC4^WT^.

### 5.4. Immunoprecipitation

Protein A/G magnetic beads (Thermo Fisher Scientific, 25 µl) were resuspended and washed in TBS-T (20 mM Tris, 150 mM NaCl + 0.05% Tween-202) before incubation with 2 µl anti-GFP (ab290, Abcam, RRID:AB_303395) for 1 h at room temperature. Whole cell lysate (2 mg) was added to the antibody–bead mixture and incubated overnight at 4°C. Beads were washed three times in TBS-T and proteins eluted in 1× sample buffer for 10 min at room temperature. The supernatant was collected and incubated at 95°C for 5 min. Total eluate was loaded for SDS-PAGE and western blotting.

### 5.5. Immunohistochemistry

Whole flies were prefixed in PFAT-DMSO (4% paraformaldehyde, PBS, 0.1% Triton X-100, 5% DMSO) for 1 h at room temperature, followed by washing and dissection in PBT (10 mM phosphate buffer, pH 7.4, 0.5% Triton X-100). For mushroom body analyses, adult flies were anaesthetized with CO_2_ and placed on ice prior to dissection. Third instar crawling larvae were washed and dissected in PBS (10 mM phosphate buffer, pH 7.4). Tissues were post-fixed in PFAT-DMSO for 20 min and stored in 100% methanol. Tissues were rehydrated in 50% methanol/PBT, blocked for 3 h at room temperature in immunobuffer (5% normal goat serum in PBT) and incubated overnight at room temperature with primary antibody (mouse anti-FasII, 1:20, DSHB clone 1D4, RRID:AB_528235; rabbit anti-GFP, 1:20 000, Abcam ab290, RRID:AB_303395; rat anti-HA, 1:500, Roche clone 3F10, RRID:AB_2687407; rabbit anti-MEF2, 1:500, gift from Bruce Paterson, National Cancer Institute, Bethesda; rabbit anti-Myc, 1:100, Abcam ab9106, RRID:AB_307014; mouse anti-Myc, 1:100, DSHB clone 9E10, RRID:AB_2266850), followed by overnight incubation at 4°C with secondary antibody (goat anti-mouse Alexa 555, 1:500, Invitrogen A-21422, RRID:AB_2535844; goat anti-rabbit Alexa 647, 1:500, Invitrogen A21244, RRID:AB_2535812; goat anti-rat Alexa 555, 1:500, Invitrogen A21434, RRID:AB_2535855), then washing in PBT. Brains were incubated with FSB (0.01% in 50% ethanol) or THT (0.25% in 50% ethanol), washed in 50% ethanol, and then washed further in PBT. Samples that required nuclear staining were washed in PBT before incubation with DAPI (1:20 000 in PBS) and all tissues were mounted in Antifade (90% glycerol, 0.2% n-propyl gallate, 10 mM phosphate buffer, pH 7.4 (Sigma Aldrich), 0.5% Triton X-100). Images were captured using a Leica TCS SP5 DM6000B confocal microscope or a Zeiss LSM900 super-resolution microscope, and processed using ImageJ software.

### 5.6. Live imaging

Live imaging of adult *Drosophila* brains was performed using a Zeiss LSM900 super-resolution microscope using a protocol modified from [70]. Brains were dissected from live flies in PBS and immediately mounted in Voltalef 10S oil, a coverslip placed atop, and imaged using a 63× oil immersion lens.

### 5.7. Light microscopy

Light microscopy was performed using an Olympus SZX16 stereo zoom microscope and CellSens Dimension (Olympus) imaging software. Flies were frozen at −20°C before thawing and imaging at 110× magnification. Constant light intensity and exposure was used. Images were imported into Adobe Photoshop (RRID:SCR_014199), Z-axis drift accounted for using the Auto-Align Layers function, and optical sections stacked using the Auto-Blend Layers function.

### 5.8. Scanning electron microscopy

Scanning electron microscopy was performed as previously described [42]. Briefly, adult flies were anaesthetized and immersed in primary modified Karnovsky’s fixative (3% glutaraldehyde, 2% formaldehyde, 0.1 M sodium phosphate buffer and Triton X-100), followed by vacuum infiltration, ethanol dehydration and critical point drying. Heads were mounted onto aluminium stubs, sputter coated with gold (Bal-Tec SCD 050 sputter coater), and imaged using an FEI Quanta 200 environmental scanning electron microscope (20.00 kV, spot 4.0 nm).

### 5.9. Quantification of condensation and neurodevelopmental phenotypes

HDAC4 and MEF2 condensates in Kenyon cell nuclei were counted using the Cell Counter plugin (https://imagej.net/ij/plugins/cell-counter.html, RRID:SCR_025376) in ImageJ and were only counted if they were visible in both single and merged channels. For panels in figures 2D and 7B and electronic supplementary material, figure S3, condensates were counted through ten serial optical sections at 1 µm increments, beginning at the first emergence of the calyx (*n* = 4 brains per genotype), imaged at 150× magnification. When condensates were present in adjacent slices they were only counted once. For panels in figures 3I and 7D, condensates per nucleus were counted in a single optical section (705× magnification) for ≥27 cells per section (*n* ≥ 3 brains per genotype) using QuPath [71] (https://qupath.github.io/, RRID:SCR_018257). Statistical significance was assessed using a one-way ANOVA with *post hoc* Tukey’s HSD test with significance set at *α* = 0.05. Condensate size was measured using QuPath, where only condensates larger than the diameter of the Airy disc (0.288 µm) were included. Statistical significance was assessed using a two-sample unpaired *t*‐test with significance set at *α* = 0.05. Condensate movement was assessed by generating maximum projection images from 50 time-lapse frames obtained over 100 s. Condensates were scored as moving if the projection lacked a single, well-defined spherical condensate, indicating blurring due to motion. Movement was confirmed by examining individual frames throughout the time series.

Assessment of mushroom body phenotypes was performed by a blinded scorer as per Tan *et al*. [35]. Z-stacks of mushroom body lobes were scored for the presence or absence of developmental defects in the α- and β lobes, including lobe thinning, absence and guidance defects, as well as β lobe fusion. Fusion was scored as mild if axons measuring less than half the width of the lobe crossed the midline of the brain, or as severe if axons measuring more than half the width of the lobe crossed the midline. Statistical significance was assessed using Fisher’s exact test.

Assessment of eye development was performed by a blinded scorer as per Tan *et al*. [35]. A scoring system was developed to assess severity of defects in morphology which considered three key facets of eye development that can be reproducibly analysed; bristle formation, ommatidia alignment, fusion and pigmentation. Each aspect was scored from 0 to 4, for a total severity score of 12 per eye, which was converted to % severity. Bristles: 0 = All bristles present and correctly placed, 1 = Most bristles correctly placed, with no more than a small number missing or extra, 2 = Moderate loss of bristles or misplaced bristles and/or large loss of bristles at edges of the eye, 3 = Few bristles, 4 = Complete absence of bristles. Ommatidia: 0 = Ommatidia correctly aligned and no fusion, 1 = Alignment of ommatidia altered but no fusion, 2 = Ommatidia alignment perturbed, altered depth of ommatidia boundary but no fusion, 3 = Ommatidia alignment perturbed and fusion, 4 = Complete absence of ommatidia (total fusion). Pigmentation: 0 = No changes in pigmentation, 1 = Small number of ommatidia missing pigment, 2 = Moderate loss of pigmentation across the eye, 3 = Pigmentation only around the edge of the eye, or in small spots across eye, 4 = No pigmentation. Where data were normally distributed statistical significance was assessed using a one-way ANOVA with *post hoc* Tukey’s HSD test with significance set at *α* = 0.05. Where data were not normally distributed, the Kruskal–Wallis test was used with significance set at *α* = 0.05, followed by a *post hoc* Dunn’s test with a Bonferroni corrected *α*.

## Data Availability

All study data have been included in the main article and in the supplementary material [72].

## References

[1] Wang WH, Cheng LC, Pan FY, Xue B, Wang D, Chen Z, Li C. 2011 Intracellular trafficking of histone deacetylase 4 regulates long‐term memory formation. Anat. Rec. **294**, 1025–1034. (10.1002/ar.21389) 21542139

[2] Kim MS, Akhtar MW, Adachi M, Mahgoub M, Bassel-Duby R, Kavalali ET, Olson EN, Monteggia LM. 2012 An essential role for histone deacetylase 4 in synaptic plasticity and memory formation. J. Neurosci. **32**, 10879–10886. (10.1523/jneurosci.2089-12.2012) 22875922 PMC3480333

[3] Sando R, Gounko N, Pieraut S, Liao L, Yates J, Maximov A. 2012 HDAC4 governs a transcriptional program essential for synaptic plasticity and memory. Cell **151**, 821–834. (10.1016/j.cell.2012.09.037) 23141539 PMC3496186

[4] Fitzsimons HL, Schwartz S, Given FM, Scott MJ. 2013 The histone deacetylase HDAC4 regulates long-term memory in Drosophila. PLoS One **8**, e83903. (10.1371/journal.pone.0083903) 24349558 PMC3857321

[5] Zhu Y *et al*. 2019 Class IIa HDACs regulate learning and memory through dynamic experience-dependent repression of transcription. Nat. Commun. **10**, 3469. (10.1038/s41467-019-11409-0) 31375688 PMC6677776

[6] Wakeling E *et al*. 2021 Missense substitutions at a conserved 14-3-3 binding site in HDAC4 cause a novel intellectual disability syndrome. Hum. Genet. Genom. Adv. **2**, 100015. (10.1016/j.xhgg.2020.100015) PMC7841527 33537682

[7] Trazzi S *et al*. 2016 HDAC4: a key factor underlying brain developmental alterations in CDKL5 disorder. Hum. Mol. Genet. **25**, 3887–3907. (10.1093/hmg/ddw231) 27466189

[8] Broide RS, Redwine JM, Aftahi N, Young W, Bloom FE, Winrow C. 2007 Distribution of histone deacetylases 1–11 in the rat brain. J. Mol. Neurosci. **31**, 47–58. (10.1007/BF02686117) 17416969

[9] Darcy MJ, Calvin K, Cavnar K, Ouimet CC. 2010 Regional and subcellular distribution of HDAC4 in mouse brain. J. Comp. Neurol. **518**, 722–740. (10.1002/cne.22241) 20034059

[10] Sjöstedt E *et al*. 2020 An atlas of the protein-coding genes in the human, pig, and mouse brain. Science **367**, eaay5947. (10.1126/science.aay5947) 32139519

[11] Davis FJ, Gupta M, Camoretti-Mercado B, Schwartz RJ, Gupta MP. 2003 Calcium/calmodulin-dependent protein kinase activates serum response factor transcription activity by its dissociation from histone deacetylase, HDAC4. J. Biol. Chem. **278**, 20047–20058. (10.1074/jbc.m209998200) 12663674

[12] Miska EA, Karlsson C, Langley E, Nielsen SJ, Pines J, Kouzarides T. 1999 HDAC4 deacetylase associates with and represses the MEF2 transcription factor. EMBO J. **18**, 5099–5107. (10.1093/emboj/18.18.5099) 10487761 PMC1171580

[13] Vega RB *et al*. 2004 Histone deacetylase 4 controls chondrocyte hypertrophy during skeletogenesis. Cell **119**, 555–566. (10.1016/j.cell.2004.10.024) 15537544

[14] Wang AH, Yang XJ. 2001 Histone deacetylase 4 possesses intrinsic nuclear import and export signals. Mol. Cell. Biol. **21**, 5992–6005. (10.1128/mcb.21.17.5992-6005.2001) 11486037 PMC87317

[15] Lahm A *et al*. 2007 Unraveling the hidden catalytic activity of vertebrate class IIa histone deacetylases. Proc. Natl Acad. Sci. USA **104**, 17335–17340. (10.1073/pnas.0706487104) 17956988 PMC2077257

[16] Fischle W, Dequiedt F, Hendzel MJ, Guenther MG, Lazar MA, Voelter W, Verdin E. 2002 Enzymatic activity associated with class II HDACs is dependent on a multiprotein complex containing HDAC3 and SMRT/N-CoR. Mol. Cell **9**, 45–57. (10.1016/s1097-2765(01)00429-4) 11804585

[17] Fischle W, Dequiedt F, Fillion M, Hendzel MJ, Voelter W, Verdin E. 2001 Human HDAC7 histone deacetylase activity is associated with HDAC3 in vivo. J. Biol. Chem. **276**, 35826–35835. (10.1074/jbc.m104935200) 11466315

[18] Huang EY, Zhang J, Miska EA, Guenther MG, Kouzarides T, Lazar MA. 2000 Nuclear receptor corepressors partner with class II histone deacetylases in a Sin3-independent repression pathway. Genes Dev. **14**, 45–54. (10.1101/gad.14.1.45) 10640275 PMC316335

[19] McKinsey TA, Zhang CL, Olson EN. 2000 Activation of the myocyte enhancer factor-2 transcription factor by calcium/calmodulin-dependent protein kinase-stimulated binding of 14-3-3 to histone deacetylase 5. Proc. Natl Acad. Sci. USA **97**, 14400–14405. (10.1073/pnas.260501497) 11114197 PMC18930

[20] Grozinger CM, Schreiber SL. 2000 Regulation of histone deacetylase 4 and 5 and transcriptional activity by 14-3-3-dependent cellular localization. Proc. Natl Acad. Sci. USA **97**, 7835–7840. (10.1073/pnas.140199597) 10869435 PMC16631

[21] Wang AH, Kruhlak MJ, Wu J, Bertos NR, Vezmar M, Posner BI, Bazett-Jones DP, Yang XJ. 2000 Regulation of histone deacetylase 4 by binding of 14-3-3 proteins. Mol. Cell. Biol. **20**, 6904–6912. (10.1128/mcb.20.18.6904-6912.2000) 10958686 PMC88766

[22] Miska EA, Langley E, Wolf D, Karlsson C, Pines J, Kouzarides T. 2001 Differential localization of HDAC4 orchestrates muscle differentiation. Nucleic Acids Res. **29**, 3439–3447. (10.1093/nar/29.16.3439) 11504882 PMC55849

[23] Borghi S, Molinari S, Razzini G, Parise F, Battini R, Ferrari S. 2001 The nuclear localization domain of the MEF2 family of transcription factors shows member-specific features and mediates the nuclear import of histone deacetylase 4. J. Cell Sci. **114**, 4477–4483. (10.1242/jcs.114.24.4477) 11792813

[24] Chawla S, Vanhoutte P, Arnold FJL, Huang CL ‐H, Bading H. 2003 Neuronal activity‐dependent nucleocytoplasmic shuttling of HDAC4 and HDAC5. J. Neurochem. **85**, 151–159. (10.1046/j.1471-4159.2003.01648.x) 12641737

[25] Chen Y, Wang Y, Modrusan Z, Sheng M, Kaminker JS. 2014 Regulation of neuronal gene expression and survival by basal NMDA receptor activity: a role for histone deacetylase 4. J. Neurosci. **34**, 15327–15339. (10.1523/jneurosci.0569-14.2014) 25392500 PMC4228135

[26] Schlumm F, Mauceri D, Freitag HE, Bading H. 2013 Nuclear calcium signaling regulates nuclear export of a subset of class IIa histone deacetylases following synaptic activity. J. Biol. Chem. **288**, 8074–8084. (10.1074/jbc.m112.432773) 23364788 PMC3605626

[27] Litke C, Bading H, Mauceri D. 2018 Histone deacetylase 4 shapes neuronal morphology via a mechanism involving regulation of expression of vascular endothelial growth factor D. J. Biol. Chem. **293**, 8196–8207. (10.1074/jbc.ra117.001613) 29632070 PMC5971441

[28] Shen X, Chen J, Li J, Kofler J, Herrup K. 2016 Neurons in vulnerable regions of the Alzheimer’s disease brain display reduced ATM signaling. eNeuro **3**, ENEURO.0124-15.2016. (10.1523/ENEURO.0124-15.2016) PMC4770009 27022623

[29] Herrup K, Li J, Chen J. 2013 The role of ATM and DNA damage in neurons: upstream and downstream connections. DNA Repair **12**, 600–604. (10.1016/j.dnarep.2013.04.012) 23680599 PMC3720697

[30] Li J, Chen J, Ricupero CL, Hart RP, Schwartz MS, Kusnecov A, Herrup K. 2012 Nuclear accumulation of HDAC4 in ATM deficiency promotes neurodegeneration in ataxia telangiectasia. Nat. Med. **18**, 783–790. (10.1038/nm.2709) 22466704 PMC3378917

[31] Colussi C, Aceto G, Ripoli C, Bertozzi A, Li Puma DD, Paccosi E, D’Ascenzo M, Grassi C. 2023 Cytoplasmic HDAC4 recovers synaptic function in the 3×Tg mouse model of Alzheimer’s disease. Neuropathol. Appl. Neurobiol. **49**, e12861. (10.1111/nan.12861) 36331820 PMC10099707

[32] Sen A, Nelson TJ, Alkon DL. 2015 ApoE4 and Aβ oligomers reduce BDNF expression via HDAC nuclear translocation. J. Neurosci. **35**, 7538–7551. (10.1523/jneurosci.0260-15.2015) 25972179 PMC6705431

[33] Wu Q, Yang X, Zhang L, Zhang Y, Feng L. 2016 Nuclear accumulation of histone deacetylase 4 (HDAC4) exerts neurotoxicity in models of Parkinson’s disease. Mol. Neurobiol. **54**, 6970–6983. (10.1007/s12035-016-0199-2) 27785754

[34] Lang C *et al*. 2019 Single-cell sequencing of iPSC-dopamine neurons reconstructs disease progression and identifies HDAC4 as a regulator of Parkinson cell phenotypes. Cell Stem Cell **24**, 93–106. (10.1016/j.stem.2018.10.023) 30503143 PMC6327112

[35] Tan WJ, Hawley HR, Wilson SJ, Fitzsimons HL. 2024 Deciphering the roles of subcellular distribution and interactions involving the MEF2 binding region, the ankyrin repeat binding motif and the catalytic site of HDAC4 in Drosophila neuronal morphogenesis. BMC Biol. **22**, 2. (10.1186/s12915-023-01800-1) 38167120 PMC10763444

[36] Guo L, Han A, Bates DL, Cao J, Chen L. 2007 Crystal structure of a conserved N-terminal domain of histone deacetylase 4 reveals functional insights into glutamine-rich domains. Proc. Natl Acad. Sci. USA **104**, 4297–4302. (10.1073/pnas.0608041104) 17360518 PMC1838596

[37] Kirsh O, Seeler JS, Pichler A, Gast A, Müller S, Miska E. 2002 The SUMO E3 ligase RanBP2 promotes modification of the HDAC4 deacetylase. EMBO J. **21**, 2682–2691. (10.1093/emboj/21.11.2682) 12032081 PMC125385

[38] Bolger TA, Zhao X, Cohen TJ, Tsai CC, Yao TP. 2007 The neurodegenerative disease protein ataxin-1 antagonizes the neuronal survival function of myocyte enhancer factor-2. J. Biol. Chem. **282**, 29186–29192. (10.1074/jbc.m704182200) 17646162

[39] Liu L *et al*. 2024 14-3-3 binding motif phosphorylation disrupts Hdac4-organized condensates to stimulate cardiac reprogramming. Cell Rep. **43**, 114054. (10.1016/j.celrep.2024.114054) 38578832 PMC11081035

[40] Nam J, Gwon Y. 2023 Neuronal biomolecular condensates and their implications in neurodegenerative diseases. Front. Aging Neurosci. **15**, 1145420. (10.3389/fnagi.2023.1145420) 37065458 PMC10102667

[41] Li F *et al*. 2020 The connectome of the adult Drosophila mushroom body provides insights into function. eLife **9**, e62576. (10.7554/elife.62576) 33315010 PMC7909955

[42] Main P, Tan WJ, Wheeler D, Fitzsimons HL. 2021 Increased abundance of nuclear HDAC4 impairs neuronal development and long-term memory. Front. Mol. Neurosci. **14**, 48. (10.3389/fnmol.2021.616642) PMC8042284 33859551

[43] Schwartz S, Truglio M, Scott MJ, Fitzsimons HL. 2016 Long-term memory in Drosophila is influenced by histone deacetylase HDAC4 interacting with SUMO-conjugating enzyme Ubc9. Genetics **203**, 1249–1264. (10.1534/genetics.115.183194) 27182943 PMC4937474

[44] Dai S *et al*. 2024 Structural insights into the HDAC4–MEF2A–DNA complex and its implication in long-range transcriptional regulation. Nucleic Acids Res. **52**, 2711–2723. (10.1093/nar/gkae036) 38281192 PMC10954479

[45] Michalik A, Van Broeckhoven C. 2003 Pathogenesis of polyglutamine disorders: aggregation revisited. Hum. Mol. Genet. **12**, R173–R186. (10.1093/hmg/ddg295) 14504263

[46] Crooks RO, Rao T, Mason JM. 2011 Truncation, randomization, and selection: generation of a reduced length c-Jun antagonist that retains high interaction stability. J. Biol. Chem. **286**, 29470–29479. (10.1074/jbc.m111.221267) 21697091 PMC3190987

[47] Brand AH, Perrimon N. 1993 Targeted gene expression as a means of altering cell fates and generating dominant phenotypes. Development **118**, 401–415. (10.1242/dev.118.2.401) 8223268

[48] Hawley HR, Roberts CJ, Fitzsimons HL. 2023 Comparison of neuronal GAL4 drivers along with the AGES (auxin-inducible gene expression system) and TARGET (temporal and regional gene expression targeting) systems for fine tuning of neuronal gene expression in Drosophila. MicroPublication Biol. (10.17912/micropub.biology.000885) PMC10314298 37396791

[49] Robinow S, White K. 1988 The locus elav of Drosophila melanogaster is expressed in neurons at all developmental stages. Dev. Biol. **126**, 294–303. (10.1016/0012-1606(88)90139-x) 3127258

[50] Connolly JB, Roberts IJH, Armstrong JD, Kaiser K, Forte M, Tully T, O’Kane CJ. 1996 Associative learning disrupted by impaired Gs signaling in Drosophila mushroom bodies. Science **274**, 2104–2107. (10.1126/science.274.5295.2104) 8953046

[51] McGuire SE, Mao Z, Davis RL. 2004 Spatiotemporal gene expression targeting with the TARGET and gene-switch systems in Drosophila. Science **2004**, 220. (10.1126/stke.2202004pl6) 14970377

[52] Chan JKL, Sun L, Yang XJ, Zhu G, Wu Z. 2003 Functional characterization of an amino-terminal region of HDAC4 that possesses MEF2 binding and transcriptional repressive activity. J. Biol. Chem. **278**, 23515–23521. (10.1074/jbc.m301922200) 12709441

[53] Juhl DW, Risør MW, Scavenius C, Rasmussen CB, Otzen D, Nielsen NC, Enghild JJ. 2019 Conservation of the amyloid interactome across diverse fibrillar structures. Sci. Rep. **9**, 3863. (10.1038/s41598-019-40483-z) 30846764 PMC6405930

[54] Banani SF, Lee HO, Hyman AA, Rosen MK. 2017 Biomolecular condensates: organizers of cellular biochemistry. Nat. Rev. Mol. Cell Biol. **18**, 285–298. (10.1038/nrm.2017.7) 28225081 PMC7434221

[55] Lee T, Lee A, Luo L. 1999 Development of the Drosophila mushroom bodies: sequential generation of three distinct types of neurons from a neuroblast. Development **126**, 4065–4076. (10.1242/dev.126.18.4065) 10457015

[56] Freymuth PS, Fitzsimons HL. 2017 The ERM protein Moesin is essential for neuronal morphogenesis and long-term memory in Drosophila. Mol. Brain **10**, 41. (10.1186/s13041-017-0322-y) 28851405 PMC5576258

[57] Duffy JB. 2002 GAL4 system in Drosophila: a fly geneticist’s Swiss army knife. Genesis **34**, 1–15. (10.1002/gene.10150) 12324939

[58] Kumar JP. 2012 Building an ommatidium one cell at a time. Dev. Dyn. **241**, 136–149. (10.1002/dvdy.23707) 22174084 PMC3427658

[59] Cagan R. 2009 Principles of Drosophila eye differentiation. Curr. Top. Dev. Biol. **89**, 115–135. (10.1016/S0070-2153(09)89005-4) 19737644 PMC2890271

[60] Cagan RL, Ready DF. 1989 The emergence of order in the Drosophila pupal retina. Dev. Biol. **136**, 346–362. (10.1016/0012-1606(89)90261-3) 2511048

[61] Freeman M. 1996 Reiterative use of the EGF receptor triggers differentiation of all cell types in the Drosophila eye. Cell **87**, 651–660. (10.1016/s0092-8674(00)81385-9) 8929534

[62] Chen S, Berthelier V, Yang W, Wetzel R. 2001 Polyglutamine aggregation behavior in vitro supports a recruitment mechanism of cytotoxicity. J. Mol. Biol. **311**, 173–182. (10.1006/jmbi.2001.4850) 11469866

[63] Alberti S, Dormann D. 2019 Liquid–liquid phase separation in disease. Annu. Rev. Genet. **53**, 171–194. (10.1146/annurev-genet-112618-043527) 31430179

[64] Schulz RA, Chromey C, Lu MF, Zhao B, Olson EN. 1996 Expression of the D-MEF2 transcription in the Drosophila brain suggests a role in neuronal cell differentiation. Oncogene **12**, 1827–1831. 8622904

[65] Backs J *et al*. 2011 Selective repression of MEF2 activity by PKA-dependent proteolysis of HDAC4. J. Cell Biol. **195**, 403–415. (10.1083/jcb.201105063) 22042619 PMC3206346

[66] Gambis A, Dourlen P, Steller H, Mollereau B. 2011 Two-color in vivo imaging of photoreceptor apoptosis and development in Drosophila. Dev. Biol. **351**, 128–134. (10.1016/j.ydbio.2010.12.040) 21215264 PMC3051417

[67] Madeira F, Madhusoodanan N, Lee J, Eusebi A, Niewielska A, Tivey ARN, Lopez R, Butcher S. 2024 The EMBL-EBI job dispatcher sequence analysis tools framework in 2024. Nucleic Acids Res. **52**, W521–W525. (10.1093/nar/gkae241) 38597606 PMC11223882

[68] Schneider CA, Rasband WS, Eliceiri KW. 2012 NIH image to ImageJ: 25 years of image analysis. Nat. Methods **9**, 671–675. (10.1038/nmeth.2089) 22930834 PMC5554542

[69] Maitra U, Scaglione MN, Chtarbanova S, O’Donnell JM. 2019 Innate immune responses to paraquat exposure in a Drosophila model of Parkinson’s disease. Sci. Rep. **9**, 12714. (10.1038/s41598-019-48977-6) 31481676 PMC6722124

[70] Savoian MS. 2017 Microscopy methods for analysis of spindle dynamics in meiotic Drosophila spermatocytes. Methods Mol. Biol. **1471**, 265–276. (10.1007/978-1-4939-6340-9_15) 28349402

[71] Bankhead P *et al*. 2017 QuPath: open source software for digital pathology image analysis. Sci. Rep. **7**, 16878. (10.1038/s41598-017-17204-5) 29203879 PMC5715110

[72] Hawley HR, Sutherland-Smith AJ, Savoian MS, Fitzsimons HL. 2025 Supplementary material from: N-terminal oligomerisation drives HDAC4 nuclear condensation and neurodevelopmental dysfunction in Drosophila. Figshare. (10.6084/m9.figshare.c.8081912)41151763

